# Functional Local Renin-Angiotensin System in Human and Rat Periodontal Tissue

**DOI:** 10.1371/journal.pone.0134601

**Published:** 2015-08-05

**Authors:** Carlos F. Santos, Ana C. Morandini, Thiago J. Dionísio, Flávio A. Faria, Marta C. Lima, Caio M. Figueiredo, Bella L. Colombini-Ishikiriama, Carla R. Sipert, Rubens P. Maciel, Ana P. Akashi, Gabriela P. Souza, Gustavo P. Garlet, Camila O. Rodini, Sandra L. Amaral, Christiane Becari, Maria C. Salgado, Eduardo B. Oliveira, Isaac Matus, Daniela N. Didier, Andrew S. Greene

**Affiliations:** 1 Department of Biological Sciences, Bauru School of Dentistry, University of São Paulo, Bauru, São Paulo, Brazil; 2 Department of Restorative Dentistry, School of Dentistry, University of São Paulo, São Paulo, São Paulo, Brazil; 3 Department of Physical Education, Science Faculty, São Paulo State University, Bauru, São Paulo, Brazil; 4 School of Medicine of Ribeirão Preto, Riberão Preto, University of São Paulo, Riberão Preto, São Paulo, Brazil; 5 Department of Physiology, Medical College of Wisconsin, Milwaukee, Wisconsin, United States of America; Max-Delbrück Center for Molecular Medicine (MDC), GERMANY

## Abstract

The initiation or progression of periodontitis might involve a local renin-angiotensin system (RAS) in periodontal tissue. The aim of this study was to further characterize the local RAS in human and rat periodontal tissues between healthy and periodontally-affected tissue. Components of the RAS were investigated using *in vitro*, *ex vivo* and *in vivo* experiments involving both human and Wistar rat periodontium. Although not upregulated when challenged with *P*. *gingivalis*-lipopolysaccharide, human gingival and periodontal ligament fibroblasts expressed RAS components. Likewise, healthy and inflamed human gingiva expressed RAS components, some of which were shown to be functional, yet no differences in expression were found between healthy and diseased gingiva. However, in inflamed tissue the immunoreactivity was greater for the AT_1_R compared to AT_2_R in fibroblasts. When compared to healthy tissue, ACE activity was increased in human gingiva from volunteers with gingivitis. Human-gingiva homogenates generated Ang II, Ang 1-9 and Ang 1-7 when incubated with precursors. In gingiva homogenates, Ang II formation from Ang I was nearly abolished only when captopril and chymostatin were combined. Ang 1-7 formation was significantly greater when human gingiva homogenates were incubated with chymostatin alone compared to incubation without any inhibitor, only captopril, or captopril and chymostatin. In rat gingiva, RAS components were also found; their expression was not different between healthy and experimentally induced periodontitis (EP) groups. However, renin inhibition (aliskiren) and an AT_1_R antagonist (losartan) significantly blocked EP-alveolar-bone loss in rats. Collectively, these data are consistent with the hypothesis that a local RAS system is not only present but is also functional in both human and rat periodontal tissue. Furthermore, blocking AT_1_R and renin can significantly prevent periodontal bone loss induced by EP in rats.

## Introduction

**Fig 1:**
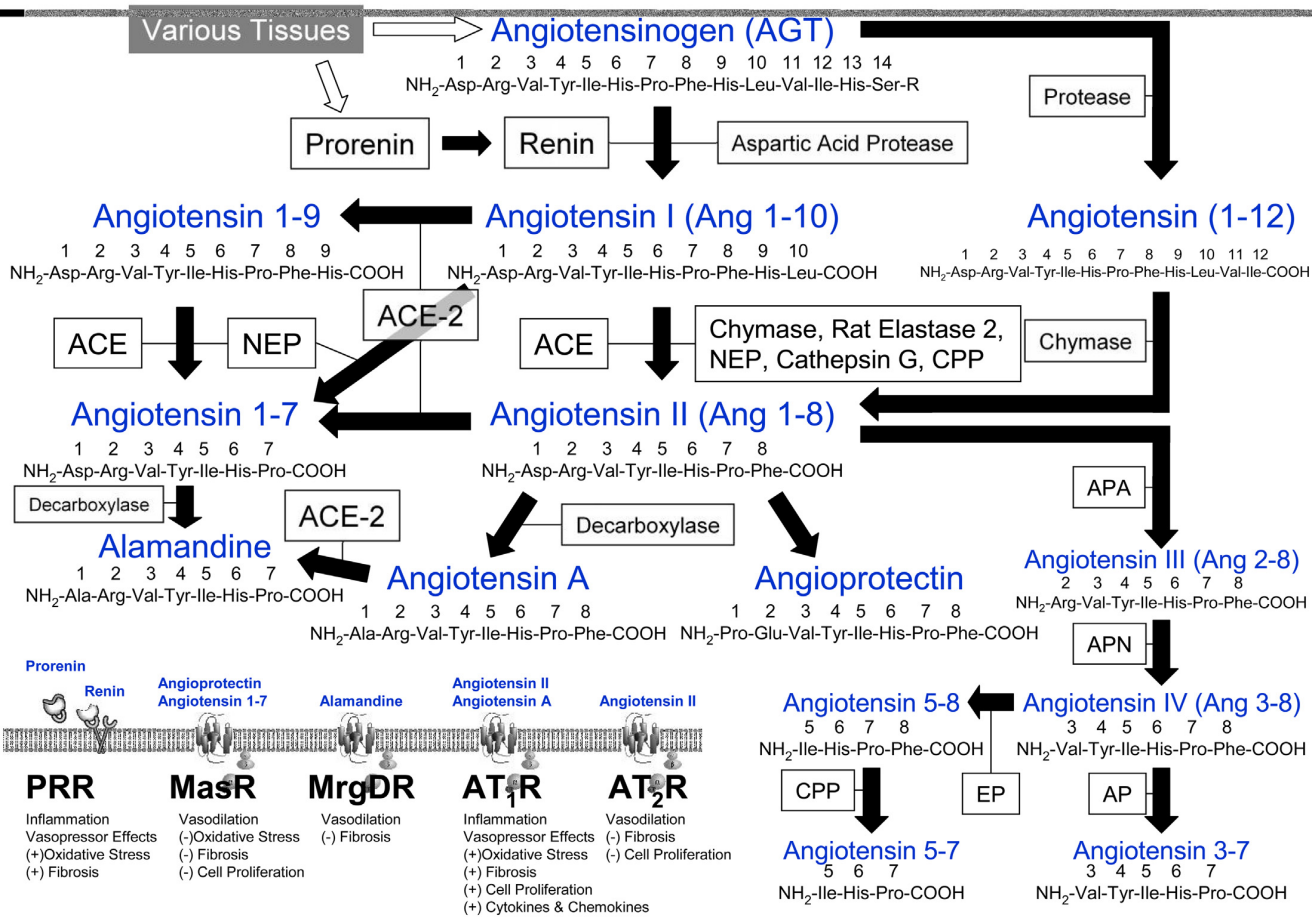
Schematic Representation of the Renin-Angiotensin System. Angiotensin-I converting enzyme, ACE; angiotensin I converting enzyme 2, ACE-2; angiotensin II 1 receptor, AT_1_R; angiotensin II 2 receptor, AT_2_R; aminopeptidase, AP; aminopeptidase A, APA; aminopeptidase N, APN; carboxypeptidase, CPP; endopeptidase, EP; Mas receptor, MasR; Mas-related gene type D receptor, MrgDR; neprilysin, NEP; (pre)prorenin receptor, PRR.

The renin-angiotensin system (RAS) regulates or modulates numerous physiological functions including blood pressure, electrolyte balance, inflammation and the release of various peptide hormones. In particular, peptides such as vasopressin and angiotensinogen (AGT) are released and transported through the circulatory system as part of the extended RAS modulating inflammation, oxidative stress, fibrosis and cell proliferation. Fig 1 depicts a broad schematic pathway of the RAS. Briefly, prorenin is converted to renin; this rate-limited step releases renin into the circulatory system thereby cleaving AGT forming angiotensin I (1–10) (Ang I). Ang I can be cleaved by angiotensin I-converting enzyme (ACE) to form angiotensin II (1–8) (Ang II) or by angiotensin I-converting enzyme 2 (ACE-2) to form angiotensin 1–9 (Ang 1–9). ACE-2 also catalyses the cleavage of Ang II to form angiotensin 1–7 (Ang 1–7). ACE can also cut Ang 1–9 to form Ang 1–7, and alamandine can be formed by Ang 1–7. Additionally, Ang II can form angiotensin III (2–8), angiotensin A and angioprotectin.

These numerous circulating peptide hormones mediate their actions by stimulating various receptors. Specifically, prorenin and renin can bind to the prorenin receptor (PRR) to stimulate inflammation, vasopressor effects, oxidative stress and fibrosis. Ang 1–7 and angioprotectin can bind to the Mas receptor (MasR) stimulating vasodilatation, inhibiting oxidative stress, fibrosis and cell proliferation. Conversely, angiotensin II 1 receptor (AT_1_R) activation by Ang II or angiotensin A, potent vasoactive peptides, stimulates oxidative stress, fibrosis, cell proliferation and a release of cytokines and chemokines which in turn mediates tissue inflammation [1]. It was further demonstrated that Ang II stimulates the proliferation of guinea pig and rabbit fibroblasts [2,3], and human gingival fibroblasts that can produce prostaglandin E_2_ (PGE_2_). PGE_2_ from gingival fibroblasts can stimulate osteoblasts to release factors that induce multiple downstream signals stimulating the resorption of bone by osteoclasts. AT_2_R activation by Ang II counters some of the effects of AT_1_R activation, namely decreasing fibrosis and cell proliferation.

Some studies have demonstrated the existence of local RAS components in oral tissue using cultured guinea pig (2002) and rabbit gingival (2004) fibroblasts and ferret gingiva (2003) *in vivo* [2,3,4]. Souza *et al*. (2007) found Ang II receptors in rat pulp tissue; these reports investigated specifically AT_1_Rs and AT_2_Rs [5]. Our group then demonstrated local RAS components in rat gingiva and found that this system was capable of generating Ang II and other vasoactive peptides *in vitro* [1].

Periodontitis, one of the most prevalent diseases worldwide, leads to gingival inflammation, alveolar bone loss and, eventually, edentulism. Approximately 100 to 200 bacterial species inhabit the average human cavity [6]. Furthermore, the teeth’s surfaces do not shed themselves, unlike the epithelial side of the periodontal pocket and thus allow the establishment of plaque biofilms that contain complex and evolving microbiota. Many factors such as environment, genetics, habits (*e*.*g*. smoking and dental hygiene), age, obesity, socioeconomic status, and transfer of species between individuals via oral contact may shift this complex of microbiota towards harmful colonies leading to periodontitis. These factors that affect the microbiota in the oral cavity might also contribute to periodontal disease directly. Additionally, a niche for pathogenic oral bacterial may form when normal oral microorganisms are absent or, as Hajishengallis *et al*. (2012) hypothesized, a few harmful bacteria might mediate a change of benign microbiota to either being harmful and/or unbeneficial [7].

The presence of certain microorganisms coincides with the onset and progression of periodontal disease. In particular, Riviere *et al*. (1996) observed increased levels of certain oral bacterial species in diseased tissue when compared to healthy tissue from humans with periodontitis [8]. Additionally, certain bacteria linked with periodontitis such as *Porphyromonas gingivalis* are significantly more numerous in deep subgingival pockets that exhibit bleeding upon probing, a clinical index of periodontal inflammation, when compared to healthy gingiva [9]. In particular, subgingival bacteria living proximal to the epithelial lining of periodontal pockets thrive on an increased flow of gingival crevice fluid enriched with degraded nutritive proteins from local inflammation [9] resulting in growth increases of these colonies in the oral cavity and periodontal pocket deepening, which may in turn increase inflammation. *P*. *gingivalis*, moreover, secretes lipopolysaccharide (LPS), an endotoxin that elicits a strong immune response by inducing the secretion of pro-inflammatory cytokines and nitric oxide in cells. However, *P*. *gingivalis* can also subvert the protective host pro-inflammatory response by direct cytokine degradation through gingipains [10] or by dampening IL-1β secretion through its fimbriae [11]. Overall the combination of the host’s background and the subgingival microbiota can lead to plaque biofilms that may release enough LPS to recruit a significant amount of leukocytes. In particular, both granulocytes and agranulocytes have been implicated in mediating local increases in proinflammatory cytokines including IL-1β, tumor necrosis factor (TNF)-α and other mediators such as prostaglandins and chemokines thereby augmenting a local inflammatory response leading to the degradation of local soft and hard tissues [12,13].

In sum, harmful microorganisms in combination with the host’s genetics, oral environment and structure can lead to changes in the immune system and possibly the RAS; in turn, the immune system and RAS influence each other which can eventually lead to gingivitis, periodontitis and possibly an increased cardiovascular risk [14]. Research continues to emerge linking the immune system with bone growth and resorption [15]. Currently, it remains uncertain if periodontal tissue contains functional or active RAS components and whether these local RAS components can, at least in part, cause, maintain or modulate periodontitis. The specific objective of this study was to evaluate the relationship between components in the RAS and other mediators with periodontitis and alveolar bone loss in humans and rats. The overarching goal was to gain a clearer understanding of the biological mechanisms involved in periodontal disease.

## Materials and Methods

### Ethical Aspects

The Ethics Committee on Human Research at the Bauru School of Dentistry—University of São Paulo (USP) (Permit Numbers: 125/2010 and 080/2008) and the Ethics Committee on Animal Research at the Bauru School of Dentistry—USP (Permit Number: 009/2008) approved these studies; additionally, these studies were executed with complete adherence to the recommendations and guidelines established by these committees. Human subjects gave written informed consent for the use of their tissue. All surgeries were performed with approved anesthetics and doses, with all efforts made to minimize suffering. Rats were euthanized with an excessive dose (60 mg/kg, intravenously) of thiopental (Thiopentax, Cristália, Campinas, SP, Brazil) to obtain samples.

### Human Volunteers

Eligible participants were healthy adults aged 20y to 60y who met the following inclusion criteria: aged >19 years with no missing teeth; volunteers with healthy gingiva with no history of inflammation, periodontitis or other dental anomalies. Exclusion criteria included the following: pregnancy, systemic illnesses, and use of illicit drugs or drugs known to affect the RAS within the last 6 mo. Thirty-four volunteers were selected from the dental clinic from USP and divided into the following three groups according to their clinical periodontal conditions: healthy gingiva (n = 12), gingivitis (n = 10) and periodontitis (n = 12). These classifications were initially based on radiographic examinations [16], plaque index [17], bleeding upon probing of the gingival sulcus, probing depths of the gingival sulcus (>5 mm), and clinical inspection [18] and then confirmed histologically (CMF).

### Experimental Animals

For experiments involving rats, 50 outbred adult-male age-matched (50 to 64 d) Wistar rats from USP weighing 196 ± 23 g to 270 ± 32 g were randomly assorted into five groups for drug treatment. More specifically, seven days prior to the start of the experiment, every rat was randomly assigned and assorted to one of five groups (two cages per group) using a blind draw of five tiles out of an opaque sack. These animals were housed (41 cm x 34 cm x 17 cm) with hardwood-shaving bedding in the same climate-controlled room (21.3 ± 0.7°C, relative humidity of 64 ± 4%) maintained on a light/dark cycle with lights on from 07:00 to 19:00 hours. Rats were acclimatized for at least 1 wk with daily handling prior to any experimental manipulation. Additionally, rats were weighed daily (13:00 to 16:00 hours) and had access to water and autoclaved food (Presence, Ratos e Camundongos, Paulínia, SP, Brazil) *ad libitum*. Drug doses, based on weight, were administered daily (13:00 to 16:00 hours) via gavage above each animal’s cage. Gavage was used since most drugs in human trails are taken orally. Injection order was achieved by randomly picking each rat out of the first randomly assigned cage, followed by the next random cage, and so forth. Half of the rats (n = 25) were used for bone loss measurements and the other half (n = 25) were used for qPCR. For bone loss experiments, an *a priori* power analysis using an effect size of 0.78 (calculated from a preliminary 7 d test) with an α error probability of 0.05 and a power of 0.80 indicated a total sample size of 25 rats (G*Power 3.1.9.2). A *post hoc* analysis with a calculated effect size of 1.0179 and an α error probability of 0.05 with a total sample size of 25 rats yielded an actual power of 0.967.

### Human Gingiva

During tooth extractions, a gingivectomy or the modified-Widman flap were employed to obtain gingiva samples (CMF). These techniques allowed the collection of two samples of marginal-gingival tissues (buccal/labial and lingual) at each of the four teeth selected for a total of eight samples of gingiva per patient. These samples were randomly coded and analyzed for the following: inflammatory cell count (IC), reverse transcription-polymerase chain reaction (RT-PCR), quantitative real-time PCR (qPCR), reverse-phase high performance liquid chromatography (HPLC), fluorimetric assay (FA), and immunohistochemistry (IHC). Following collection, gingival samples were stored in tubes with TRIzol (Life Technologies—Thermo Fisher Scientific, Waltham, Massachusetts, U.S.A.) for RT-PCR and qPCR, and other tubes for HPLC and FA at -80°C until analyzed. Additionally, samples for IC and IHC were stored at 4°C in 10% formaldehyde solution >48 h prior to processing.

#### Histological Processing of Human Tissue Samples

After fixation, samples were blocked and embedded in paraffin (CMF). Sections 5 μm thick were cut and then stained with hematoxylin and eosin (HE) for quantifying inflammatory cells, and sections 4 μm thick were stored for IHC [19,20] resulting in mesial-distal cuts which were adhered to glass slides (Corning Life Sciences, Tewksbury, MA, U.S.A.) for microscopy (COR).

#### Inflammatory Cell Counting (IC) of Human Tissue Samples

To validate the clinical classifications of each individual, inflammatory cells, lymphocytes and plasmocytes, were counted as previously described [21] for four sections with each of the two gingival specimens collected per patient. Two blinded examiners (CMF and RPM) counted cells in a 850 μm^2^ area per section containing all conjunctive tissue surrounded by gingival epithelial tissues (AxioSkop microscope, 40x objective lens, 8x ocular lens with 10 x 10 square matrix; AxioCam camera; RHc; Zeiss, Oberkochen, Germany).

#### Human Gingiva qPCR

For qPCR experiments, gingival samples were homogenized (Potter S tissue homogenizer; B. Braun Biotech International—Sartorius AG; Göttingen, Germany) and total RNA was isolated as previously described [22]. The quantity and purity of RNA extracted for reverse transcription (RT) was analyzed using a spectrophotometer (Nanodrop 1000; Thermo Fisher Scientific; Waltham, Massachusetts, U.S.A.). RNA was treated with DNAse (gDNA wipeout; Qiagen; Düsseldorf, Germany) and processed using the Quantitect RT Kit (Qiagen). After RT, the samples were quantified for the mRNAs of various RAS components using the Taqman system for qPCR (ViiA 7 qPCR machine, Life Technologies). The 20x FAM and MGB dye labeled probe-primer mix (Table 1) and the 2x Taqman Gene Expression Master Mix were used (Applied Biosystems). All qPCR methods for human gingivae were performed by two blinded researchers (TJD and DTB).

**Table 1:** Catalog numbers for primers used for human qPCR—RAS genes assays (Applied Biosystems).

Target	Source	Catalog Nunber
Angiotensin converting enzyme (ACE)	Applied Biosystems	Hs01104600_m1
Angiotensin converting enzyme 2 (ACE-2)	Applied Biosystems	Hs00222343_m1
Angiotensin II receptor type 1 (AT_1_R)	Applied Biosystems	Hs01096942_m1
Angiotensin II receptor type 2 (AT_2_R)	Applied Biosystems	Hs00169126_m1
Angiotensinogen (AGT)	Applied Biosystems	Hs01586213_m1
Mas receptor (MasR)	Applied Biosystems	Hs00364818_g1
Renin	Applied Biosystems	Hs00982555_m1
60S ribosomal protein L13a (RPLA13A)	Applied Biosystems	Hs00204173_m1

A Viia 7 qPCR machine (Life Technologies) was used to quantify mRNA with cycles consisting of a 2 min period at 50°C first, then 10 min at 95°C, followed by 50 cycles of 15 s at 95°C and 1 min at 60°C. Templates, data collection and partial data analysis was performed using Viia7 software version 1.1 (Applied Biosystems). Briefly, mRNA expression of all the target genes were normalized to the RPL13 reference gene and based on the cycle threshold (Ct) determined in part by the negative control for each set of primers and probes used. Primers used for the human qPCR study for each target along with their source and catalog numbers are listed in Table 1. The mean Ct values of duplicate measurements were used to calculate the expression of the target genes, with the normalization formula (1+efficiency)^-ΔΔCt^, assessed via manufacturer (Life Technologies) inventoried primers.

#### Whole Mount IHC with Human Tissue

Six specimens were selected from the gingiva collected from each of the three experimental groups (healthy, gingivitis and periodontitis) to be used for IHC. The IHC protocol used was previously described by Oliveira *et al*. (2008) [20]. One blinded researcher (CMF) performed all IHC for human samples. Briefly, tissue sections were deparaffinized in xylene and hydrated through graded concentrations of alcohol. Endogenous peroxidase activity was blocked using 3% hydrogen peroxide solution in methanol (0.01 M) for 30 min. Sections were heated in a microwave for two cycles of 15 min each in citrate buffer. After 20 min of cooling, the slides were rinsed with phosphate buffered saline (PBS) and nonspecific binding was blocked using a proprietary blocking solution (S0809, Dako Corporation, Carpinteria, CA, U.S.A.) for 30 min.

The antibodies used for IHC along with their respective concentrations are listed in Table 2. Primary antibodies were diluted with PBS with 1% bolvine serum albumin (pH 7.3) and placed on top of each section for 1 h at 21°C. Next the sections were incubated with biotinylated swine anti-rabbit IgG secondary antibodies (Universal Imuno peroxidase Polymer, Histofine Simple Stain Max PO (G), Nichirei, Tokyo, Japan; AT1R: anti-rabbit, catalog number 414151F) for 15 min. Sections were then rinsed and incubated with streptavidin-biotinylated horseradish peroxidase complex (Dakocytomation LSAB2 Kit) for 15 min. Peroxidase activity was visualized by incubating 3,3’-diaminobenzidine tetrahydrochloride (DAB) solution containing 1 mL/L hydrogen peroxide with the samples for 3 min. Then these sections were rinsed and counterstained with Harris’ hematoxylin solution for 2.5 min. Normal mouse IgG substituted the primary antibody to serve as a negative control. For a positive control for the immunostaining AT_1_R, human heart tissue was used for the AT_1_R [23]. For a positive control for the AT_2_R, kidney was used [24]. Sections were mounted and examined (microscope: AxioSkop; camera: AxioCam RHc; Zeiss, Oberkochen, Germany).

**Table 2:** The source and concentration of antibodies used for the human immunohistochemistry study.

Target Protein	Source	Catalog Number	Antibody	Concentration
Angiotensin II receptor type 1 (AT_1_R)	Santa Cruz Biotechnology	sc-1173	1° antibody (rabbit polyclonal IgG)	1:200
Angiotensin II receptor type 2 (AT_2_R)	Santa Cruz Biotechnology	sc-9040	1° antibody (rabbit polyclonal IgG)	1:100
anti-Rabbit	Nichirei Corporation	414151F	2° antibody (biotinylated swine Anti-Rabbit IgG)	1:100

Counting the total number of cells, inflammatory infiltrated type cells, fibroblast type cells and endothelial cells of the connective tissue was performed by three independent and blinded researchers (CMF, COR and GPG) according to the protocol indicated by Faustino et al. (2008) [25]. The numbers of positive and negative cells were counted, and the positive stained cells over the total cells ratio for each of the three cell types was calculated.

#### ACE Activity Measurement for Human Studies

ACE activity was measured using a FA for gingiva homogenates according to previous studies [1,26]. Briefly, gingiva samples were thawed, weighed, and homogenized (Potter S tissue homogenizer, B. Braun Biotech International) centrifuged (Hermle Z 326K microcentrifuge, Wehingen, Germany) and the supernatants removed and kept at 4°C until assayed for ACE activity.

ACE activity was determined using the Hippuryl-Histidine-Leucine (Hip-His-Leu) as substrate and tested measuring the dipeptide (His-Leu) formed using a FA [27]. Samples were incubated with a Hip-His-Leu solution (5mM) in Tris acetate buffered saline (TBS). After 30 min the enzymatic action was interrupted. The dipeptide His-Leu was detected by the addition of the 100 μl of 1% *o*-phthalaldehyde (mass/volume, in ethanol), followed by the addition of 200 μL of 6 M HCl after 4 min. Fluorescence was measured (Shimadzu RF-535 spectrofluorimeter, Kyoto, Japan) at 365 nm wavelength using a 495 nm emission wavelength. A standard curve for the dipeptide His-Leu (0 to 20 nmol) was used to calculate the concentration of His-Leu. ACE activity was corrected by weight (mg) for each gingiva sample. The ACE and enzymatic activity measurements in human gingiva were performed by four blinded researchers (CMF, TJD, DTB and CB).

#### Enzymatic Activity in Human Gingiva via HPLC

Five samples of gingival homogenates from each of the three experimental groups were selected for HPLC to detect in vitro enzymatic activity of Ang I and Ang II. Briefly, Ang I and Ang II were incubated with and without pharmacological inhibition via 10μM of captopril (ACE inhibitor) and/or 100μM chymostatin (serine protease inhibitor). Gingival samples were thawed, weighed, and homogenized in TBS. After centrifuge, supernatants (40 μL) were collected and incubated with 30 nmol of Ang I or Ang II in TBS, for a final volume of 150 μL for 20 min at 37°C. Cleavages in both peptides were evaluated (Shimadzu SCL-6B HPLC machine, Kyoto, Japan) coupled with a Shim-pak ODS column (4 x 250 mm). The peptides were eluted with a linear gradient of acetonitrile (10 to 32%, 30 min) in 0.1% trifluoroacetic acid (TFA) at a flow rate of 1.0 mL/min and monitored using a wavelength absorbance of 215 nm. The molar concentration of the peptides formed was calculated using a standard curve with known amounts of a cognate synthetic peptide [1,28].

### Human Fibroblasts Cell Culture

Fibroblasts were isolated and cultured from gingival and periodontal ligaments from three systemically healthy donors (2 female and 1 male, 16 to 26y old) as previously described by Morandini et al. (2010) [29]. Briefly, human periodontal ligament fibroblasts (HPLF) were collected from tissue from the middle third of extracted third molar roots. Concurrently, gingival biopsies from clinically non-inflamed tissue were retrieved from the gingival margin from the same donors. Next, cells were obtained by explants from gingival and periodontal ligament and cultivated in Dulbecco’s Modified Eagle’s Medium (DMEM, Gibco—Life Technologies—Thermo Fisher Scientific), supplemented with 10% fetal bovine serum (FBS, Gibco) and antibiotics (100 UI/mL penicillin/streptomycin, Invitrogen—Life Technologies—Thermo Fisher Scientific) and were used between the 4th and 8th passages for all the comparative analyses. Human fibroblast cell culture protocols were performed by two blinded researchers (BLC-I and ACM).

#### Immunofluorescence Staining

Human gingival fibroblasts (HGF) and HPLF from the same donors were cultured on 8-well chambered slides (0.7 cm^2^ cultured area) at a density of 10^4^ cells per well. After attachment overnight, cells were stimulated by lipopolysaccharide (LPS) from *P*. *gingivalis* or *E*.*coli* (10 μg/mL) for 24 h. Purified LPS from *P*. *gingivalis* (catalog # tlrl-pglps) and *E*.*coli* (catalog # tlrl-eklps) from InvivoGen (San Diego, CA, U.S.A.) were used. Cell culture medium was used as a negative control. After fixation with 4% paraformaldehyde for 15 min, the cells were incubated with 3% bovine serum albumin (BSA) for 30 min at 21°C, then incubated with diluted primary antibody (1:100) anti-fibroblast surface protein (FSP, Abcam) or with a mouse immunoglobulin (IgG1) as a negative control overnight at 40°C, and then finally incubated with fluorescein-conjugated secondary antibody (1:400, Abcam PLC, Cambridge, U.K.) at 37°C for 1 h in the dark. Afterwards, the slides were mounted with a mounting medium containing 4',6-diamidino-2-phenylindole (DAPI, Vector Laboratories) and then analyzed (Leica TCS SPE confocal laser scanning microscope, Mannheim, Germany). Immunofluorescence staining was performed by two blinded researchers (BLC-I and ACM).

### Periodontal Disease Induction in Rats

The experimental periodontitis (EP) model used in rats was described previously [20]. Briefly, rats were anesthetized using an intraperitoneal injection of thiopental (60 mg/Kg), secured on a surgery platform and had a silk ligature tied around the lower first molar in a submarginal position to induce EP. In a different group of rats, a sham-periodontitis-induction surgery (sham group) was also performed in parallel mimicking the periodontitis-induction surgery without the implementation of the silk ligature. After 14 d of oral administration of vehicle (sham and EP rats), aliskiren (30 mg/kg), enalapril (10 mg/kg) or losartan (50 mg/kg) rats were sacrificed. Gingival biopsies and mandibles samples were processed for IHC, bone loss and qPCR.

#### Effectiveness of Drug Tests

The drug doses used were reported effective in previous studies [30,31,32]. To test the effectiveness of enalapril and losartan, animals had a cannula implanted in the left carotid artery (for direct recording of blood pressure) and the right jugular vein for injecting drugs intravenously. Blood pressure was recorded, and the effects of intravenous injection of Angiotensin I (0.1 ng to 1 ng) and angiotensin II (10 ng to 100 ng) on blood pressure during the drugs’ peak plasma concentrations were analyzed (S1 Fig). Two researchers (TJD and SLA) completed these tests.

qPCR of Rat Gingiva. Total RNA was isolated from gingival tissues using the same protocol used for the human studies. Additionally, mRNA expression of RAS genes using qPCR were performed as previously described, using 20x FAM and MGB dye labeled probe-primer mixes (Applied Biosystems, Table 3) and β-Actin as a reference gene. All qPCR methods for rat gingivae were performed by two blinded researchers (TJD and DTB).

**Table 3:** Catalog numbers for primers used for rat qPCR—RAS genes assays (Applied Biosystems).

Target	Source	Catalog Number
Angiotensin converting enzyme (ACE)	Applied Biosystems	Rn00561094_m1
Angiotensin converting enzyme 2 (ACE-2)	Applied Biosystems	Rn01416289_m1
Angiotensin II receptor type 1a (AT_1a_R)	Applied Biosystems	Rn002758772_s1
Angiotensin II receptor type 1b (AT_1b_R)	Applied Biosystems	Rn002132799_s1
Angiotensin II receptor type 2 (AT_2_R)	Applied Biosystems	Rn00560677_s1
Angiotensinogen (AGT)	Applied Biosystems	Rn00593114_m1
Mas receptor (MasR)	Applied Biosystems	Rn00562673_s1
Renin	Applied Biosystems	Rn00561847_m1
β-actin	Applied Biosystems	Rn00667869_m1

#### Detecting and Localizing Renin, ACE, AT_1_R and AT_2_R using IHC

IHC analysis was performed with minor modifications as previously described [20]. Mandibular with gingiva samples (n = 2 animals without periodontal disease induction and n = 5 animals in which periodontal disease was induced) were harvested and fixed on 10% formaldehyde solution for 48 h. Samples were routinely processed for paraffin embedding in blocks of ~8 mm x 10 mm x 6 mm. Sections 5 μm thick were obtained and attached to slides for immunohistochemical analysis. Tissue sections were deparaffinized, and the activity of endogenous peroxidase was blocked using 0.3% hydrogen peroxide solution in methanol (0.01 M) for 30 min. Non-specific binding was blocked by incubating the sections for 30 min with blocking solution (H-3401, Vector Laboratories, Burlingame, CA, U.S.A.). Sections were incubated with primary goat polyclonal anti-renin antibody (sc 27318, Santa Cruz Biotechnology, Santa Cruz, CA, U.S.A.) or goat polyclonal anti-ACE antibody (sc 12187, Santa Cruz Biotechnology, Santa Cruz, CA, U.S.A.) or rabbit polyclonal anti-AT_1_ (sc 1173, Santa Cruz Biotechnology, Santa Cruz, CA, U.S.A.) antibody or rabbit polyclonal anti-AT_2_ antibody (sc 9040, Santa Cruz Biotechnology, Santa Cruz, CA, U.S.A.), all antibodies diluted 1:200 in TBS (0.03 M Tris-HCl containing 0.15 M NaCl, pH 8.1) containing 1% horse serum (S-200, Vector Laboratories, Burlingame, CA, U.S.A.) for 60 min at 4°C. The sections were then rinsed with TBS and incubated with biotinylated horse anti-goat secondary antibody (BA-9500, Vector Laboratories, Burlingame, CA, U.S.A.) diluted 1:100 in TBS–1% horse serum for 30 min at 21°C. Sections were rinsed in TBS containing 0.1% Triton X-100 (Bio-Rad, Hercules, CA, U.S.A.) and incubated with the ABC reagent for 30 min at 21°C (PK-6200, Vector Laboratories, Burlingame, CA, U.S.A.). Peroxidase activity was visualized with 3,3'-diaminobenzidine (DAB, SK-4100, Vector Laboratories Burlingame, CA, U.S.A.) solution containing 1 ml/L hydrogen peroxide solution for 3 min under light protection. Reaction was halted by rinses with distilled water, and the slides were counterstained with hematoxylin (S0809, Dako, Carpinteria, CA, U.S.A.) for 2 min at 21°C according to the manufacturer’s instructions. The primary antibodies were not used in negative controls. Positive controls were performed on paraffin-embedded longitudinal sections of rat renal cortex for Renin and ACE and longitudinal sections of rat adrenal gland for AT_1_R and AT_2_Rs. Detection of RAS components using IHC was completed by three blinded researchers (CMF, GPG and COR). Additionally, inflammatory cells were counted and represented by one blinded researcher (COR) who scored the inflammation of rats with 14 d of EP. Scores ranged from one through four and were based on inflammatory infiltrate, junctional epithelium, cementum and alveolar bone crest analysis (S2 Fig).

#### Bone Loss Measurements in Rats

After 14 d of EP, rats had their mandibles excised and the gingivae were removed and flash frozen in liquid nitrogen for qPCR. Half of the mandibles were placed in 100 mL of a freshly prepared solution of 0.1 M dihydrate ethylenediaminetetraacetic acid with 0.1 M of NaOH in ddH_2_0 every week for 4 wk for histological analysis, and the other half were placed in H_2_O_2_ with daily changes of fresh H_2_0_2_ for 1 wk for bone-loss measurements. These mandibles were then stained with methylene blue (3.1 mM) for 30 s. Once dry, mandibles were uniformly mounted on a platform with a scale bar and photographed (AM4115ZT USB digital microscope, Dino-Lite Edge, New Taipei City, Taiwan). These images were captured using proprietary software bundled with the digital camera (DinoCapture 2.0 version 1.5.4, New Taipei City, Taiwan). For an indication of bone loss, the area between the cementoenamel junction (CEJ) and the alveolar bone crest (ABC) proximal to the first molar was measured (mm^2^) using ImageJ (ImageJ 1.48v, Bethesda, Maryland, U.S.A.) by three independent and blinded researchers (ACM, DTB and TJD).

#### Histological Processing of Rat Tissue Samples

Histological processing of the rat tissue was prepared using the same methods described above.

### Statistics

Statistical analysis was performed (DTB and TJD) using IBM SPSS statistics (version 20). Briefly, all data were tested for normal distribution using the Shapiro-Wilk test. Interexaminer reliability and intraexaminer reliability were evaluated using the Cohen’s kappa coefficient. Data normally distributed were presented as a means (±1 SD) and analyzed using analysis of variance followed by Tukey’s test for multiple comparisons or using Student’s *t*-test when comparing two independent groups. Kruskal–Wallis one-way analysis of variance was used for analyzing non-parametric data and presented as a median (±1 quartile). The Friedman test was used to detect differences among the volunteers. Statistical significance was set at 5%.

## Results

### RAS Component Expression in Healthy and Inflamed Human Gingiva

Initially each volunteer’s gingiva was evaluated clinically. Histological counts of inflammatory cells validated the clinical classification of the three experimental groups (health, gingivitis and periodontitis). Furthermore, histological analysis indicated that the number of plasmocytes were significantly increased in the gingiva of donors classified as having gingivitis (60±43) when compared to the number of plasmocytes in the gingiva from healthy donors (7±4, data not shown).

**Fig 2:**
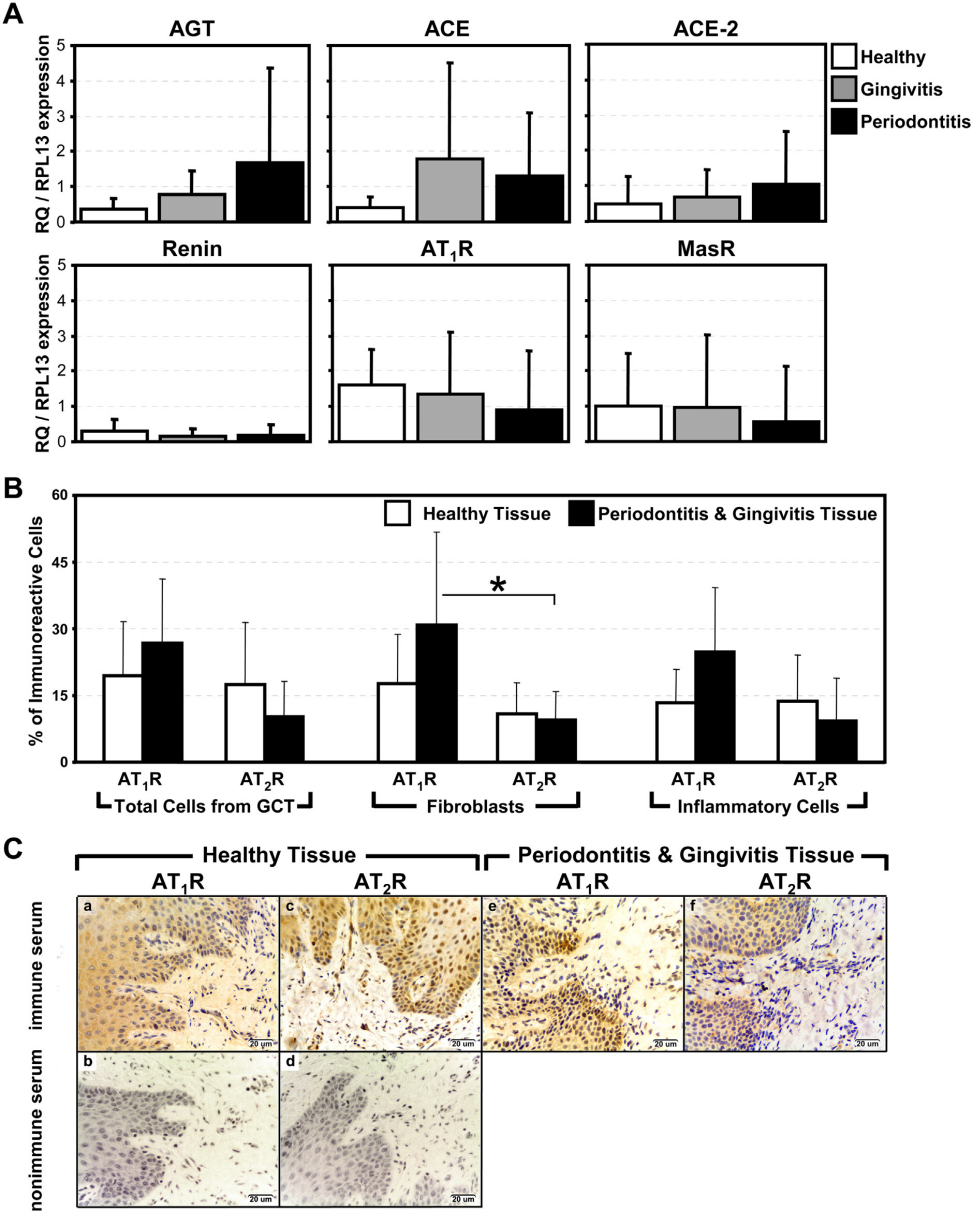
Renin-Angiotensin System Components in Human Gingiva. **A)** qPCR analysis of the following RAS components extracted from human gingiva from healthy (open bars), gingivitis (gray bars) and periodontitis (black bars) groups: angiotensinogen (AGT), angiotensin converting enzyme (ACE), angiotensin converting enzyme 2 (ACE-2), renin, angiotensin II receptor type 1 (AT_1_R), and Mas receptor (MasR), (n = 7). Graphs displays relative expression levels of the target mRNA relative to RPL-13 mRNA. The means were compared using a 1-way ANOVA and Tukey’s test. **B)** Immunoreactivity of AT_1_R and AT_2_R in the gingiva from healthy volunteers (open bars) and volunteers with gingivitis and periodontitis combined (black bars). The means were compared using a 1-way ANOVA and Ang with gingivitis and periodontitis is indicated by *. **C)** Representative photographs of immunoreactivity for either AT_1_R (Ca and Ce) or AT_2_R (Ce and Cf) in the gingiva from either healthy volunteers (Ca and Cd) or volunteers with gingivitis and periodontitis (Ce and Cf). Photographs Cb and Cd are from tissue incubated with nonimmune serum in Healthy Tissue. Scale bars indicate a distance of 20μm.

The mRNAs for RAS components (AGT, renin, ACE, ACE-2, AT_1_R, and MasR) were expressed in the gingiva of volunteers from all three groups. No differences in the expression of mRNAs among all three groups were found when compared within each mRNA target (Fig 2A). In particular, the mRNA expression for the AT_1_R in gingiva was not different among the healthy, gingivitis and periodontitis groups 1.6 (±1.0), 1.4 (±1.7) and 0.9 (±1.7) respectively; likewise the mRNA expression for the MasR was not different among the gingiva from the healthy group, gingivitis group and periodontitis group, 1.0 (±1.5), 1.0 (±2.1) and 0.6 (±1.6) respectively (Fig 2A). Additionally, among each group of volunteers no differences were found in the expression of the mRNAs for AGT, ACE, ACE-2 and renin (Fig 2A).

Human gingiva from healthy donors and donors classified as having gingivitis and periodontitis were analyzed for the presence and localization of various RAS proteins using IHC. It was found that connective tissue, fibroblasts, inflammatory cells and endothelial cells in the gingiva from all volunteer groups had positive immunoreactivity for both AT_1_Rs and AT_2_Rs (Fig 2B and 2C). Moreover, the immunoreactivity for AT_1_Rs and AT_2_Rs in healthy tissue was not significantly different among the cell types tested (connective tissue, fibroblasts and inflammatory cells). However, the immunoreactivity for AT_1_Rs in fibroblasts was greater when compared to immunoreactivity for AT_2_Rs in fibroblasts (*p*-value < 0.05, Fig 2B).

### ACE Activity in Human Gingiva

**Fig 3:**
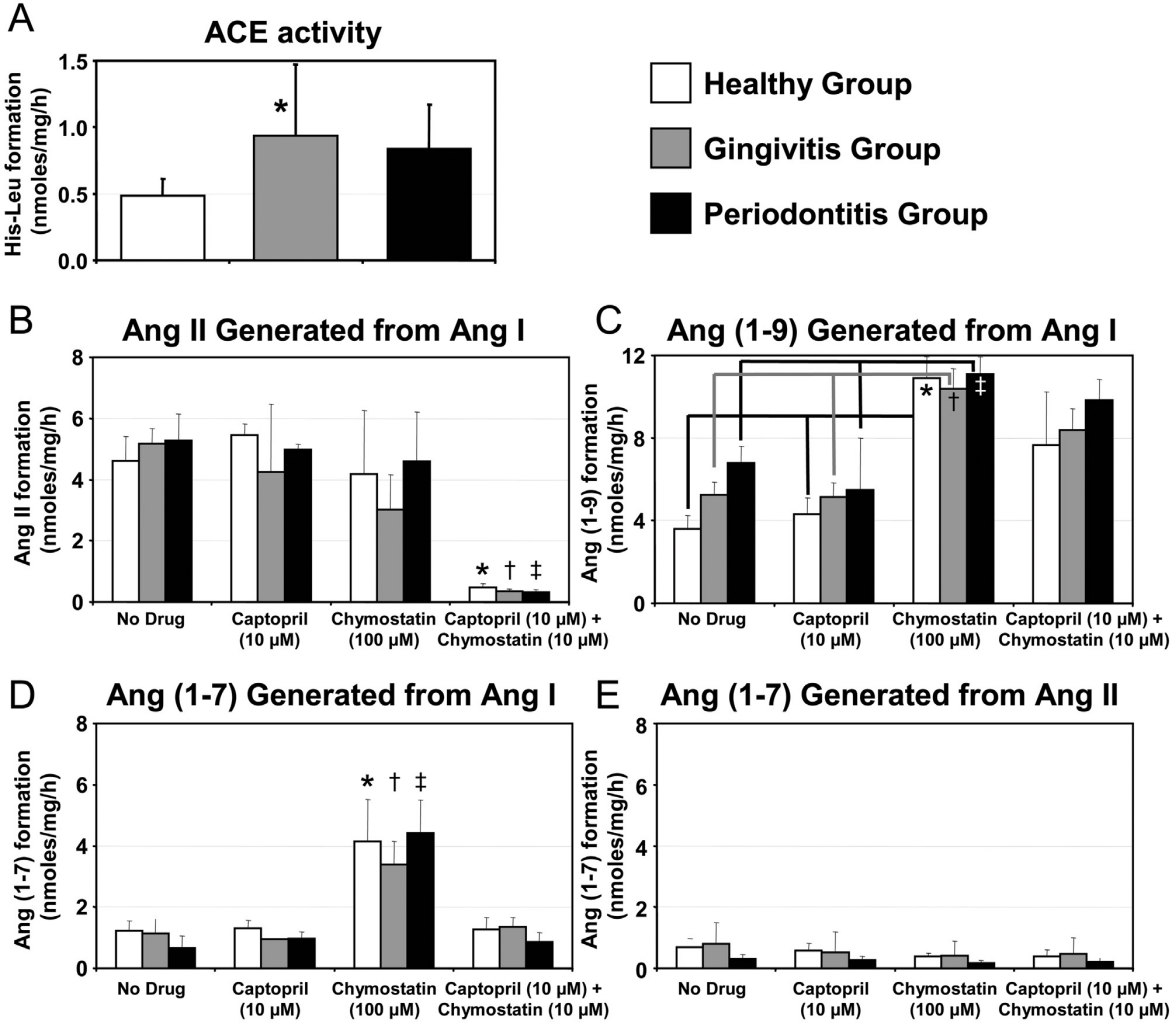
ACE Activity and Generation of Renin-Angiotensin System Components from Human Gingiva. **A)** Fluorimetric assay of ACE activity in gingiva homogenates from donors with healthy gingiva (open bar), gingivitis (gray bar) or periodontitis (black bar). The means were compared using a 1-way ANOVA and Tukey’s test. * indicates significant difference from healthy group. **B to E)** HPLC assay of homogenates from all three groups after incubation with either captopril (10μM), chymostatin (100μM), both captopril (10μM) and chymostatin (100μM), or nothing. * indicates significant differences from all other healthy groups (open bars); † indicates significant differences from all other gingivitis groups (gray bars); ‡ indicates significant differences from all other periodontitis groups (black bars); unless otherwise noted with brackets (*e*.*g*. Fig 3C). **B)** Indicates the amount of Ang II formed when incubated with Ang I. **C)** Indicates the amount of Ang 1–9 formed when incubated with Ang I. **D)** Indicates the amount of Ang 1–7 formed when incubated with Ang I. **E)** Indicates the amount of Ang 1–7 formed when incubated with Ang II.

Since no differences were observed between the mRNA of AT_1_Rs and AT_2_Rs between healthy and diseased tissue, the activity of ACE and RAS components were measured in the healthy, gingivitis and periodontitis groups. Fluorometric analysis indicated increased ACE activity in gingiva samples from volunteers with gingivitis compared to the healthy group (*p*-value = 0.02, Fig 3A). However, gingivae from volunteers with periodontitis had moderate ACE activity and were not significantly different from either healthy or gingiva homogenates (*p*-value > 0.05).

Gingiva homogenates from the healthy, gingivitis and periodontitis groups all generated Ang II, Ang 1–9 and Ang 1–7 when incubated with a precursor (Ang I or Ang II) as determined by HPLC (Fig 3B to 3E), indicating that ACE, ACE-2, and other enzymes that form Ang II were present and functional. Ang II formation was decreased (*p*-value < 0.05) when gingiva homogenates were incubated with both captopril and chymostatin when compared to incubation without any inhibitor or only captopril or chymostatin (Fig 3B). Ang 1–9 formation was significantly greater when gingiva homogenates were incubated with chymostatin when compared to incubation without any inhibitor or only captopril (Fig 3C). Conversely, Ang 1–7 formation was significantly greater when gingiva homogenates were incubated with chymostatin compared to incubation without any inhibitor, only captopril, or captopril and chymostatin (Fig 3D). Ang 1–7 generated from Ang II was nearly equivalent with Ang 1–7 generated from Ang I, but homogenates incubated with chymostatin were no different from all other conditions (Fig 3E). No differences were observed among the healthy, gingivitis and periodontitis groups in the generation of Ang II, Ang 1–9 or Ang 1–7.

### Human Gingival and Periodontal Ligament Fibroblasts Produce RAS Components

**Fig 4:**
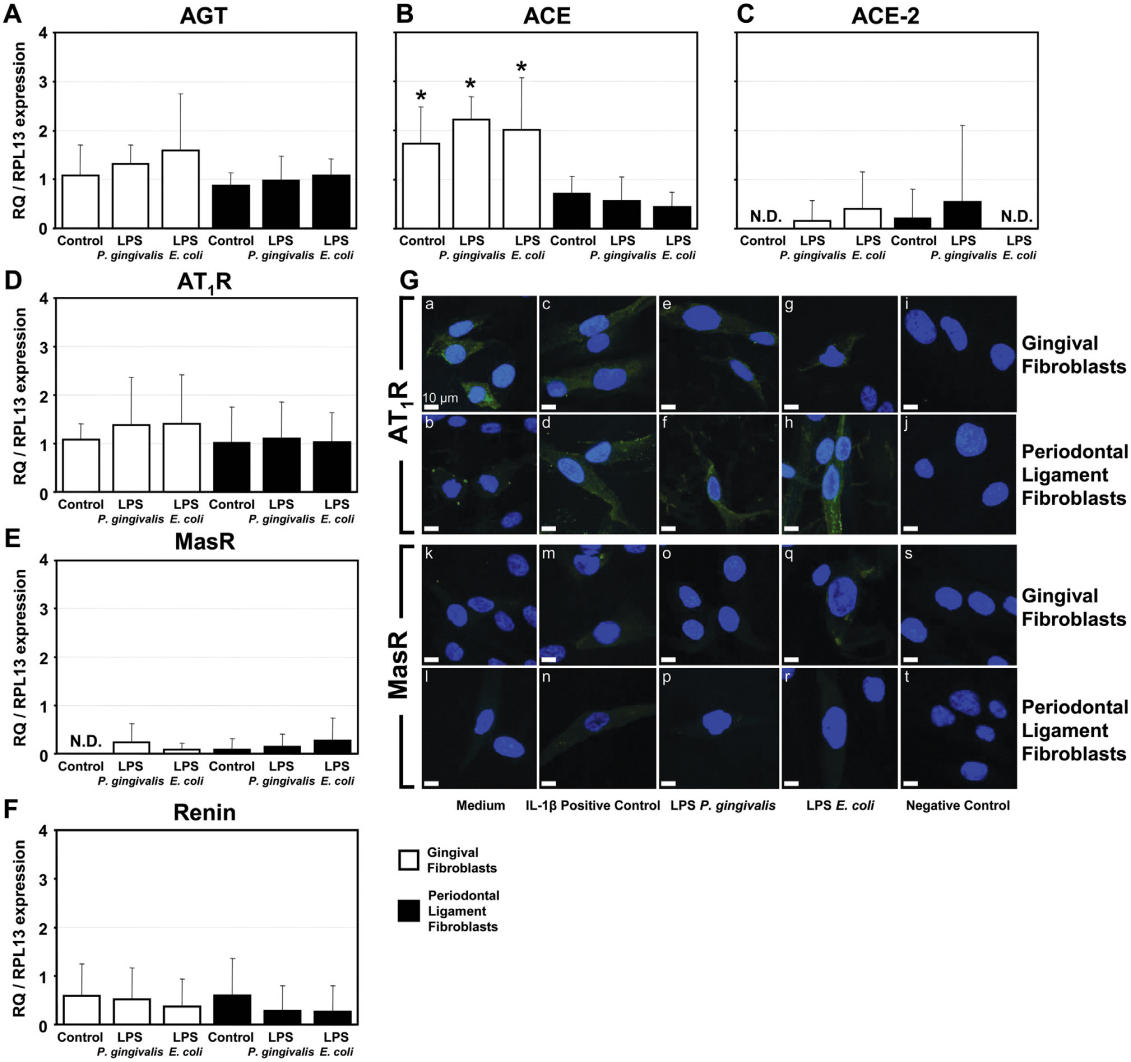
Renin-Angiotensin System Components in Human Fibroblasts. **A to F)** qPCR analysis of the following RAS components from human cultured gingival fibroblasts and periodontal ligament fibroblasts stimulated without or with LPS from *P*. *gingivalis* or *E*. *coli*: **A)** angiotensinogen (AGT), **B)** angiotensin converting enzyme (ACE), **C)** angiotensin converting enzyme 2 (ACE-2), **D)** angiotensin II receptor type 1 (AT_1_R), **E)** Mas receptor (MasR), and **F)** renin. Graphs displays relative expression levels of the target mRNA relative to RPL-13 mRNA from 3 donors in triplicate (n = 3). The means of gingival fibroblasts (open bars) and periodontal ligament fibroblasts (black bars) were compared using a 1-way ANOVA and Tukey’s test. N.D. = not detected. **G)** Fluorescent immunoreactivity of AT_1_R and MasR in gingival fibroblasts or periodontal ligament fibroblasts (portrayed in rows) when incubated with either cell culture medium as a negative control, interleukin-1β (IL-1β) as a positive control, lipopolysaccharide (LPS) from *P*. *gingivalis*, LPS from *E*. *coli*, or tissue without LPS as a negative control (portrayed in columns).

Beyond the demonstration of functional local RAS components in human gingiva, both cultured human gingival fibroblasts (GFs) and periodontal ligament fibroblasts (PLFs) expressed some components of the RAS under normal conditions and when stimulated with *P*. *gingivalis* and *E*. *coli* LPS. As indicated in Fig 4A–4F, the mRNA for AGT, ACE, ACE-2, AT_1_R, MasR and renin were detected in both GFs and PLFs, yet only the quantity of ACE mRNAs were significantly different between the fibroblast subtypes having greater expression in GFs when compared to PLFs (Fig 4B). Fibroblasts stimulated with LPS (10 μg/mL/24 h) did not alter the expression of RAS components compared to control (Fig 4A–4F). AT_2_R mRNA expression was found in fibroblasts stimulated with LPS but results were variable.

Punctated positive-immunoreactivity for the AT_1_R was frequently and consistently observed on both GFs and PLFs with and without LPS stimulation from *P*. *gingivalis* and *E*. *coli* (Fig 4G panels a to h). In contrast, some limited immunoreactivity for the MasR was found in some GFs with and without LPS stimulation from *E*. *coli* (Fig 4G panels k to r). No immunoreactivity was found in all negative controls (Fig 4G panels i, j, s and t).

### RAS Components in Rat Gingiva

**Fig 5:**
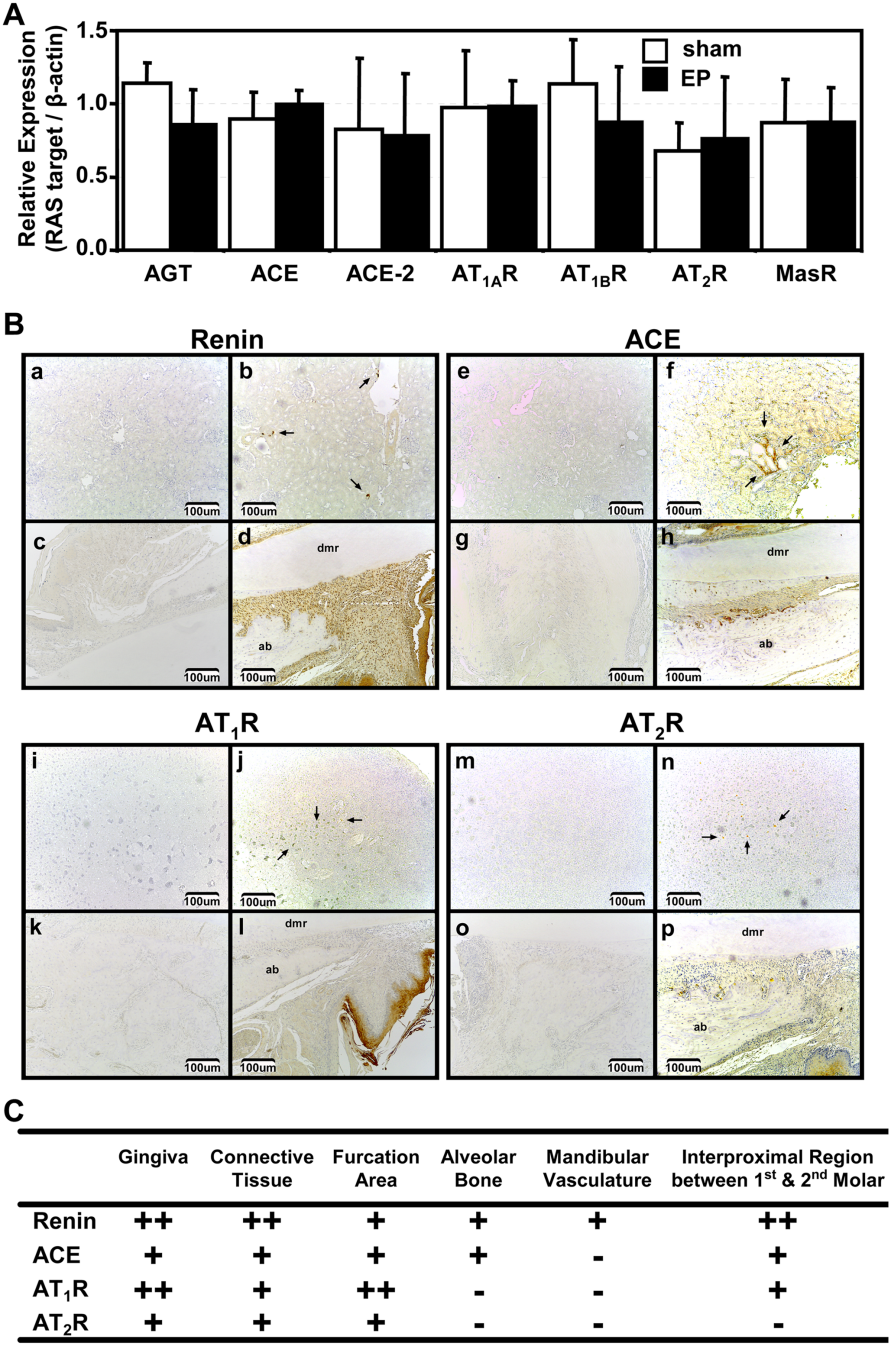
Renin-Angiotensin System Components in the Gingiva Tissue of Rats with Experimentally Induced Periodontitis. **A)** Results of qPCR analysis for mRNA of various RAS components extracted from the rat gingiva of either sham surgery (sham) or experimentally induced periodontitis (EP) for 14 days; tested RAS components included the following: angiotensinogen (AGT), angiotensin converting enzyme (ACE), angiotensin converting enzyme 2 (ACE-2), angiotensin II receptor type 1A (AT_1A_R), angiotensin II receptor type 1B (AT_1B_R), angiotensin II receptor type 2 (AT_2_R), and Mas receptor (MasR). Graph displays expression levels of the target mRNA relative to β-actin mRNA from 5 rats in duplicate (n = 5). Solid bars represent the means with SD of sham group; whereas, open bars represent animals with EP. A one-way ANOVA and Tukey’s test with statistical significance set at *p*-value < 0.05 was used. **B)** All images are at 10x magnification and scale bars indicate a distance of 100 μm. Immunoreactivity (IR) for renin (panels a to d), ACE (panels e to h), AT_1_R (panels i to l) or AT_2_R (panels m to p) in rat gingiva and bone tissue. Brown staining indicates positive IR. Black arrows indicate some of the positive IR in the positive controls. Negative **(panel a)** and positive **(panel b)** control for renin in rat kidney. **Panel c)** negative control for renin in mandible of rat with 14 d of EP treated with water. **Panel d)** immunoreactivity for renin in mandible of rat with 14 d of EP treated with water. Negative **(panel e)** and positive **(panel f)** control for ACE in a rat kidney. **Panel g)** negative control for ACE in mandible of rat with 14 d of EP treated with water. **Panel h)** immunoreactivity for ACE in mandible of rat with 14 d of EP treated with water. Negative **(panel i)** and positive **(panel j)** control for AT_1_Rs in a rat adrenal gland. **Panel k)** negative control for AT_1_Rs in mandible of rat with 14 d of EP treated with water. **Panel l)** Immunoreactivity for AT_1_Rs in mandible of rat with 14 d of EP treated with water. Negative **(panel m)** and positive **(panel n)** control for AT_2_Rs in a rat adrenal gland. **Panel o)** negative control for AT_2_Rs in mandible of rat with 14 d of EP treated with water. **Panel p)** immunoreactivity for AT_2_Rs in mandible of rat with 14 d of EP treated with water. **C)** Table indicating location of immunoreactivity observed in 2 sections of 5 rats with 14 d of EP treated with water for renin, ACE, AT_1_Rs and AT_2_Rs in different mandibular regions: (-) indicates negative immunoreactivity, (+) indicates positive immunoreactivity and (++) indicates abundant immunoreactivity.

An animal model was used with pharmacological manipulation of the RAS. Analysis using qPCR indicated that mRNA expression levels of the RAS components tested (AGT, ACE, ACE-2, AT_1A_R, AT_1B_R, AT_2_R and the MasR) were present but not significantly different between rats with and without EP after 14d (Fig 5A). Additionally, renin mRNA expression levels were undetectable by qPCR in either group of rats. Ancillary experiments indicated that enalapril (10 mg/Kg) and losartan (50 mg/Kg) doses used were physiologically effective, since blood pressure remained steady after injections of Ang I or Ang II (S1B and S1C Fig, respectively). Additionally, the dose used for aliskiren (30 mg/Kg) was shown to be physiologically effective since these treated rats had decreased basal arterial blood pressure when compared to rats not treated with aliskiren (S1 Fig).

The gingival tissue of rats with (n = 5) and without (n = 5) EP for 14 d given only water were also analyzed for the presence of mRNA of various RAS components. The mRNAs of AGT, ACE, ACE-2, AT_1A_R, AT_1B_R, AT_2_R and the MasR were present in both sham rats and rats with EP; however, no significant differences were detected between these two sets of rats (Fig 5A). Moreover, the mRNA expression for these RAS components was investigated in rats also treated with aliskiren (30 mg/kg), losartan (50 mg/kg) and enalapril (10 mg/kg), and no differences were found among all groups, including water and sham with two exceptions (S3 Fig). First, the relative quantity of mRNA for the AT_1A_R for rats treated with enalapril was significantly increased when compared to the losartan group, and, second, the relative quantity of mRNA for the AT_2_R for rats treated with losartan was significantly increased when compared to all other groups (S3 Fig).

The mandibles of rats with (n = 5) and without (n = 5) EP for 14 d given only water were also analyzed for the presence and localization of various RAS proteins using IHC. Specifically, water-treated rats with 14d of EP had positive immunoreactivity (IR) in various regions of their mandibles for the four RAS components investigated (renin, ACE, AT1R and AT2R), see Fig 5B. The IR for renin was found on fibroblasts, osteoclasts and osteoblasts, and renin IR was denser near and adjacent to bone resorption areas on alveolar bone surrounding the third molar (Fig 5Bd), yet also found throughout the mandible (Fig 5Bd and 5C). Numerous osteoclasts and a few fibroblasts adjacent to the bone resorption area on the alveolar bone surface facing the third molar roots had positive IR for ACE (Fig 5Bh); additionally, pockets of ACE IR were located throughout the mandible excluding the mandibular vasculature, as reported in Fig 5C. Other ACE immunoreactive fibroblasts not only adjacent to the surface of the alveolar bone were also abundant. Positive IR for the AT_1_R and the AT_2_R were both found in gingiva, connective tissue and the furcation area proximal to the third mandibular molar (Fig 5B and 5C). Moreover, in the gingiva of rats (with EP for 14 d given only water) AT_1_R IR was denser when compared to AT_2_R IR as demonstrated by Fig 5Bl and 5Bp and reported in Fig 5C. AT_1_R IR and AT_2_R IR, to some extent, were commonly found on epithelial cells on the superficial layer of gingiva above the alveolar bone crest proximal the third molar (Fig 5Bl and 5Bp, respectively). Whereas the interproximal region between the alveolar bone and the third molar root had limited AT_1_R and AT_2_R IR (Fig 5Bl and 5Bp, respectively). The furcation area next to the third molar had positive IR for both AT_1_Rs and AT_2_Rs (Fig 5C). Lastly, no immunoreactivity was found in negative controls for the IHC of the RAS components in the rats studied (Fig 5B, panels a, e, i, and m) whereas positive controls all had immunoreactivity (Fig 5B, panels b, f, j, and n).

**Fig 6:**
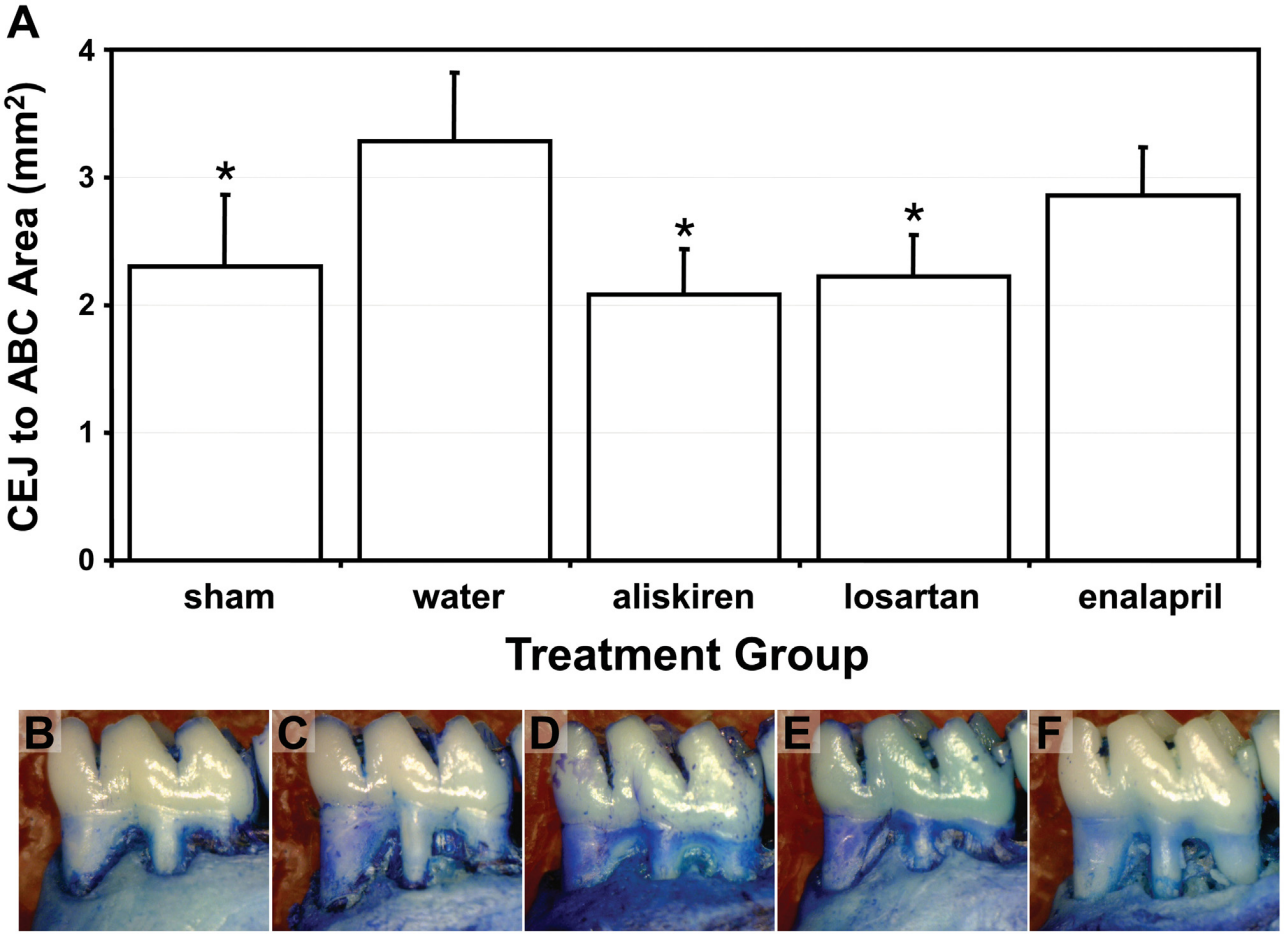
Bone Loss in Rats with Experimentally Induced Periodontitis. **A)** Graph with representative images indicating the amount of area measured between the cementoenamel junction (CEJ) to the alveolar bone crest (ABC) of the first molar after 14 d of experimentally induced periodontal disease (EP) or fictitious induction (sham). Rats were treated with vehicle (water), losartan (50 mg/kg), aliskiren (30 mg/kg) or enalapril (10 mg/kg). All groups n = 5. Statistical significance was determined by ANOVA with interaction analysis and Tukey’s test. Statistical significance (*p*-value < 0.05) is indicated by * *vs*. periodontal disease rats treated with water. **B to F)** Representative images of each of the following: sham group treated with water **(B)**, EP group treated with water **(C)**, aliskiren **(D)**, losartan **(E**) or enalapril **(F**). The samples pictured have the following respective CEJ to ABC area: (B) 2.1 mm^2^, (C) 3.3 mm^2^, (D) 2.1 mm^2^, (E) 2.1 mm^2^, (F) 3.1 mm^2^.

Since the mRNAs and proteins of RAS components were demonstrated in rat periodontal tissue, pharmacological agents were used to block different components of the RAS *in vivo*. EP rats (14d) had significant bone loss proximal to the first molar region when treated with vehicle compared to sham rats given vehicle (Fig 6). Notably, treatment with losartan (*p*-value = 0.03) and aliskiren (*p*-value = 0.01) for 14d after EP prevented alveolar-bone loss when compared to EP rats given only vehicle (Fig 6). Enalapril treatment had no effect on alveolar-bone loss (Fig 6).

## Discussion

The renin-angiotensin system (RAS) modulates inflammation in a variety of clinical conditions including cancer (Regulska et al, 2013), diabetes (Rahimi et al, 2014) and rheumatoid arthritis [33]. Moreover, in 2009 Santos *et al*. demonstrated the presence of some local RAS components in the rat gingiva providing a possible connection between periodontal disorders and the RAS [1]. This possible connection was further investigated in human and rat models by analyzing whether these components were expressed and/or functional in human gingiva derived from patients diagnosed with gingivitis and periodontitis and rats with experimentally induced periodontitis (EP). Both qPCR and IHC experiments analyzing the presence of RAS components confirmed the presences of RAS components in human gingival tissue and rat mandibles after 14 d of EP. This study not only confirmed the presence of mRNA for the classic RAS components in both human and rat gingiva (via PCR), but also demonstrated for the first time mRNA expression for angiotensin converting enzyme 2 (ACE-2) and the Mas receptor (MasR). Additionally, this study also demonstrated the expression and function of RAS components in human inflamed periodontal tissue.

RAS components were observed in human gingiva, yet no observed differences were found in the mRNA expression and protein localization of the angiotensin II receptors, AT_1_R and AT_2_R, when comparing healthy gingiva to gingiva from patients with periodontitis. AT_1_Rs were more expressed in various cell types including fibroblasts when compared to AT_2_R expression, but increased levels of local expression do not necessarily represent increased function in tissue. Thus angiotensin-I converting enzyme (ACE) activity was also investigated, one of the principal enzymes involved in angiotensin II (Ang II) generation from angiotensin I (Ang I). ACE activity was significantly increased in samples obtained from humans with gingivitis compared to healthy individuals. In contrast, the presence of periodontitis did not increase ACE activity. Thus the conversion of Ang I to Ang II induced by ACE could be more important in the initiation and progression of healthy tissue to gingivitis and/or gingivitis to periodontitis rather than the maintenance of chronic periodontitis. Indeed, the classical actions of Ang II are usually attributed to the interaction with the AT_1_R, as reviewed elsewhere [34].

In the current study, animals with EP were treated with an exact dose of enalapril (10 mg/kg, via gavage) for 14 d. However, Gonçalvez-Zillo et al. (2013), who studied bone loss in rats and mice with EP for 21 d, reported that the ACE inhibitor enalapril (via drinking water) at both a hypotensive dose (60 mg/L) and normotensive dose (12 mg/L) decreased bone loss in rats when compared to control animals with EP given water without enalapril [35]. Perhaps a time and/or dose dependent effect exists for ACE inhibition with enalapril, warranting further investigation.

Even with increased ACE activity in the gingivitis group when compared to the healthy or periodontitis groups, the biochemical analysis of Ang II generated from Ang I indicated no difference among the three human groups, which might be explained by the possible degradation of Ang II by the time it was evaluated. Moreover, ACE inhibition via captopril alone was unable to reduce the generation of Ang II from Ang I in all the groups, perhaps due to the presence of other proteases such as neprilysin (NEP), Cathepsin G, carboxyl peptidases (CPP), rat elastase-2 and other chymases, which are also capable of converting Ang I to Ang II [36,37,38]. To test this supposition, chymostatin, a serine protease inhibitor, was used to treat human gingival tissue and the same profile of Ang II formation was observed compared to ACE inhibition via captopril. In gingiva homogenates, Ang II formation from Ang I was nearly abolished only when captopril and chymostatin were combined (Fig 3B). This result suggests that both ACE and chymostatin-sensitive enzymes are involved in converting Ang I to Ang II in human gingiva and that these enzymes are both endogenous and functional. Stated in other words, when only one endogenous functional enzyme is blocked then the other enzymes can still generate Ang II from Ang I in human gingiva homogenates (Fig 3B). A similar mechanism might explain why an ACE inhibitor (enalapril) alone would not inhibit alveolar bone loss in the rats tested *in vivo* (Fig 6).

In the human gingiva homogenates, some Ang 1–7 can be generated from Ang I and Ang II and ACE-2 regardless of captopril and chymostatin (Fig 3D and 3E, respectively). In cell culture, Rice et al. (2004) showed that functional ACE-2 has much greater catalytic efficiency for converting Ang 1–7 from Ang II (2.2x10^6^M^-1^s^-1^) when compared to the reaction which converts Ang I to Ang 1–9 via ACE-2 (3.3x10^4^M^-1^s^-1^) [36]. If this result can be fairly applied to this study, then it seems reasonable to assume that the reactions for generating Ang 1–7 occur more efficiently along the Ang I—Ang II—Ang 1–7 axis then the Ang I—Ang 1–9 Ang 1–7 axis. Additionally, captopril should eliminate the degradation of Ang 1–7 to Ang 1–5 but not affect the Ang 1–7 conversion to alamandine (Fig 3E). The elimination of Ang 1–7 via conversion to alamandine seemed to be unaffected by either captopril or chymostatin or the combination of both. Therefore, the significant increase in Ang 1–7 when treated with chymostatin only (Fig 3D) does not seem to be a function of downstream events but rather from upstream events. Since significantly more Ang 1–9 was created from Ang I, the effects seen in the increase in Ang 1–7 may simply be due to the same chymostatin sensitive mechanism involved in the generation of Ang 1–9.

In the human gingiva homogenates, it was expected that captopril and chymostatin combined would effectively block Ang II production providing more substrate (Ang I) for ACE-2 to create Ang 1–9 while also limiting the production of Ang 1–7 from Ang 1–9, yet an abundance of Ang 1–9 was not observed with captopril and chymostatin combined (Fig 3C). It is thus speculated that chymostatin does not effectively block all functional enzymes (*e*.*g*. NEP) and thus some Ang 1–9 is converted to Ang 1–7 via one of these uninhibited enzymes in the human gingiva homogenates. However it remains unknown what chymostatin sensitive enzyme is affecting the quantity of Ang 1–9. Additionally, the experimental data depicted in Fig 3C provides some indirect evidence that functional endogenous ACE-2 exists since Ang 1–9 was produced by Ang I.

The involvement of fibroblasts from both gingiva and periodontal ligaments as the predominant sentinel cell type in periodontium was then investigated. AGT, renin, ACE-2, AT_1_R and the MasR each had low mRNA expression with no differences among constitutive cells or cells challenged with LPS from *P*. *gingivalis* or *E*. *coli*. ACE was the only target that was significantly more expressed in gingival fibroblasts compared to periodontal ligament cells regardless of the group tested, further supporting the hypothesis that RAS may be involved in the progression of gingivitis rather then the maintenance.

In the present study it was found that ACE, AT_1_R, AT_2_R and renin were expressed in different regions of the oral microenvironment (*e*.*g*. apical portion of the root, interdental papilla, and oral epithelium) which suggests that the pleiotropic distribution of RAS components in the periodontal tissue of rats may be indicative of distributed function. Notably, the renin-inhibitor aliskiren and AT_1_R-inhibitor losartan were able to prevent bone loss in rats when treated daily for 14d after induction of experimental periodontitis (EP). Similarly, Suda *et al*., using a mouse model, found that the AT_1_R-blocker telmisartan prevented alveolar-bone loss in mice heterozygous for FBN1 (Marfan syndrome) infected with *P*. *gingivalis* for 14 d when compared to control mice which were heterozygous for FBN1 without *P*. *gingivalis* infection [39]. Additionally, Araújo *et al*., using rats, found that telmisartan (10 mg/kg) prevented alveolar bone loss in rats with 11 d of EP via ligature when compared to sham rats [40]. Although speculative, local inflammation and other downstream effects from the AT_1_R could lead to bone loss. It follows that decreased local inflammation could decrease gingival crevice fluid, in turn decreasing nutrients for subgingival microorganisms including *P*. *gingivalis* and other critical microorganisms. LPS or other destructive factors would then be diminished, inhibiting bone loss or the development of periodontitis. Upregulation of AT_2_R mRNA was observed in rats with 14 d of EP treated with losartan when compared to all other rat groups (S3 Fig). Although speculative, it is possible that blocking the AT_1_R with losartan causes the free endogenous Ang II to stimulate the AT_2_R [41] which in turn might stimulate AT_2_R transcription.

No differences in mRNAs of AT_1_Rs and MasRs were observed in humans and rats between healthy and diseased gingivae, and no differences in the immunoreactivity for the proteins of AT_1_Rs and MasRs were observed among the human groups tested. However, it is possible that the activity and functional location of these receptors might be different. Additionally, it might be possible that an AT_1_R antagonist is somehow directly dampening bacteria invasion or destruction of the periodontal tissue. In support of this idea, a recent study using another competitive inhibitor for the AT_1_R (telmisartan) in a newborn mouse model of meningitis reported that the AT_1_R directly associates with an *E*. *coli* outer membrane receptor indicating that *E*. *coli* invasion may depend partly on the AT_1_R. Moreover, this study reported that the outer membrane receptor of *E*. *coli* can induce the formation of a protein complex involving AT_1_R and the toll-like receptor 2 (TLR2) which could mediate bacteria invasion [42]. For this reason, it is possible that some similar mechanisms occur during experimental periodontitis *in vivo*.

When rats were treated with the ACE inhibitor enalapril, bone loss was not prevented suggesting that alternative pathways in the RAS could be modulating bone loss. As aforementioned, ACE inhibition alone did not reduce Ang II formation in human gingiva. Additionally, bradykinin (BK) can induce bone resorption by stimulating osteoblasts and the cells that surrounds bone, such as gingival fibroblasts, to produce pro-inflammatory mediators such as prostaglandin E_2_ (PGE_2_), known to induce bone resorption [43]. It has been previously demonstrated that ACE inhibitors increase the probability of acute gout inflammation induced by monosodium urate [44], which was reported to enhance B1R signaling [45]. Notably, numerous people are treated with ACE inhibitors to combat cardiovascular and renal diseases which can exist simultaneously with periodontitis. These results are relevant since bone loss is a hallmark response of periodontitis in humans.

RANKL, RANK and OPG have numerous common regulators including interleukin 6 (IL-6) [46], transforming growth factor alpha (TGFβ) [47,48] and tumor necrosis factor (TNF) [49]. A local immune response arising from periodontal pathogens can trigger lymphocytes to express RANKL [50]. In particular, osteoblasts can be stimulated to produce RANKL in various ways including by its IL1β receptor via IL1β indirectly from periodontal pathogens mediated by activated immune cells such as CD4^+^ T cells. In one study, mice after oral inoculation of *Aggregatibacter actinomycetemcomitans* developed functionally active CD4^+^ T cells (but not CD8^+^ T cells or B cells) that produced RANKL in periodontal tissue which the authors report triggered alveolar bone destruction [51]. Another group investigating patients with periodontitis found that both T and B cells expressed functional type II membrane-bound RANKL (mRANKL) and membrane-free/soluble RANKL (sRANKL) [52]. Another group using a mouse model found that *P*. *gingivalis* induced RANKL expression in both T cells (CD3^+^) and B cells (CD19^+^) which the authors suggest can mediate bone resorption independent of osteoblasts [53]. T cells may also indirectly modulate RANKL by stimulating RANKL production in osteoblasts via IL-1, IL-11 and TNFα [49]. Fibroblasts also have also been shown to release both RANKL [54] and OPG [55].

Moreover, mRANKL via a juxtacrine mechanism can activate preosteoclasts, yet the metalloprotease-disintegrin TNFα converting enzyme (TACE) released by T cells can free mRANKL in osteoblasts, PD fibroblasts and lymphocytes allowing sRANKL to activate preosteoclasts via a paracrine action if uninhibited by OPG [56]. Notably, Nakashima et al (2000) suggest that mRANKL may be more effective when compared to unbound RANKL [57] while Mizuno et al (2002) found the converse [58]. With TACE, T cells have yet another way to indirectly regulate the RANKL-RANK axis. In human gingival samples from healthy donors and individuals with gingivitis and periodontitis, Bostanci et al (2008) found that TACE levels positively correlated with levels of RANKL and were significantly increased in individuals with periodontitis when compared to healthy gingiva and patients with gingivitis [59].

**Fig 7:**
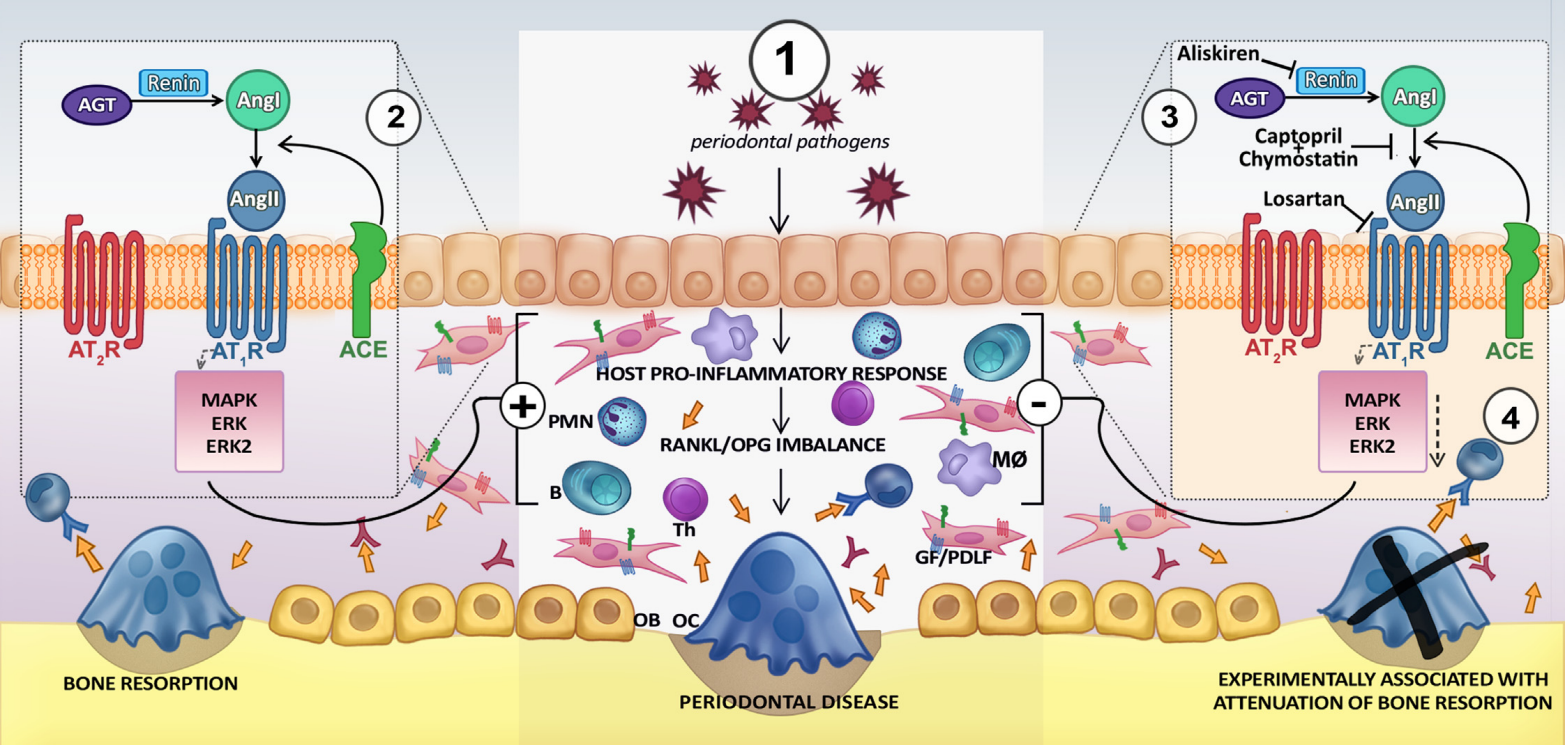
Scheme of Possible Mechanisms of the Renin-Angiotensin System (RAS) in Periodontal Tissue. **1**) Periodontal pathogens induce a host-pro-inflammatory response which can affect the RANK-RANKL-OPG axis in bone metabolism. RANKL/OPG balance is an important factor for regulating bone resorption in the periodontal environment. Osteoclast differentiation and activation are driven by the interaction of RANK (receptor activator of nuclear factor-кB) with its ligand, RANKL. Osteoprotegerin (OPG) is a decoy receptor for RANKL that inhibits RANK-RANKL engagement, as previously reviewed by Graves et al. (2011) [70]. **2**) AT_1_R and AT_2_R are present in periodontal tissue, including periodontal fibroblasts. Ang I is generated from angiotensinogen (AGT) by renin. Ang II generated from Ang I (by ACE) acts via the AT_1_R to induce the activation of the ERK pathway which in turn can upregulate the expression of RANKL in osteoblasts leading to a variety of cellular outcomes [60,67] and possibly enhancing bone resorption. **3**) Ang II formation decreased when human gingiva homogenates were incubated with both captopril (ACE inhibitor) and chymostatin. After 14 days of experimentally-induced periodontitis, renin inhibition by treatment with aliskiren or an AT_1_R antagonist (*e*.*g*. losartan) significantly attenuated bone resorption (**4**), probably decreasing activation of AT_1_R downstream pathway.

The AT_1_R localized on osteoblasts [60,61] may trigger reactive oxygen species (ROS), affecting RAS and subsequently extracellular-signal-regulated kinase (ERK) and P38 mitogen-activated protein kinase (p38) which both modulate NF-кB leading to various factors such as osteocalcin (OCN), alkaline phosphatase (ALP), IL-1, IL-6, TNFα, MMPs, and RANKL.[60] By inhibiting the AT_1_Rs on osteoblasts either directly with an AT_1_R antagonist (*e*.*g*. losartan) or upstream of Ang II by inhibiting the production of the Ang II precursors by blocking renin (*e*.*g*. aliskiren) thereby inhibiting the production of IL-1β, NF-кB, TNFα, ALP and ERK; crucial for osteoblasts’ production of RANKL (IL-1β,); cell differentiation of preosteoblasts into osteoblasts (TNFα); and the matrix calcification by mature osteoblasts (ALP). This possible mechanism is illustrated in Fig 7. It is important to note that this hypothesized AT_1_R mechanisms is not suggested to be responsible for all periodontal bone loss.

Interestingly, there may be RANKL-RANK independent axis [62,63] that stimulates the proliferation and/or survival of osteoclasts independent of TNF receptor associated factor 6 (TRAF6) [63] triggered by TNFα which may be still be dependent on CSF1. Izu et al (2008) found that both osteoclasts and osteoblasts expressed AT_1_Rs and AT_2_Rs in mice tibia cells, and that bone cells expressed renin and ACE [64]. Additionally, the bone mass of mice treated with an AT_1_R antagonist (losartan, 14 d) were no different from control (PBS) whereas mice treated with an AT_2_R antagonist (PD123319, 14 d) had increased bone mass compared to control (PBS). Thus it is implied that AT_2_R stimulation would enhance bone resorption. Monnouchi et al (2010 & 2011) also found that mice tibia cells and human PD fibroblasts stretch-loaded decreased RANKL expression only in response to an AT_2_R antagonist (PD123319) and not with the AT_1_R antagonist candesartan further corroborating research by Izu et al.(2008) [64,65,66].

In contrast, various studies find no difference between AT_2_R antagonists and control, but find a difference between the AT_1_R antagonists and control [60,67]. Shimizu et al (2008) using a estrogen-deficient rat model and cultured rabbit bone cells found Ang II stimulated RANKL in osteoblasts and TRAP^+^ cells respectively, which was abolished with an AT_1_R antagonist (olmesartan), but not with an AT_2_R antagonist (PD123329) [67]. In rats with ligature-induced EP, Araújo et al (2013a and 2013b) found that the AT_1_R antagonist olmesartan at a moderate dose (6 mg/kg), but not at a lower or higher dose, and telmisartan (10 mg/kg) reduced IL-1β, RANK, RANKL TNFα and COX-2, and increased OPG when compared to control animals given only vehicle [40,68]. This finding corroborates the proposed mechanisms speculated above for the AT_1_R antagonist (losartan) used in this study. Lastly, in mice, Asaba et al. (2009) found functional AT_1_Rs on osteoblasts, and preosteoclasts but not on mature osteoclasts; whereas they found functional AT_2_Rs on osteoblasts but not on preosteoclasts or mature osteoclasts [69].

In summary, observations were consistent with the hypothesis that a local RAS system in gingiva not only exists but is also functional in both human and rat periodontal tissue. Furthermore, antagonizing the AT_1_R and renin can significantly prevent periodontal bone loss induced by EP in rats. Further investigations might elucidate the exact mechanisms of this interplay between RAS components and periodontal inflammation thereby extending the therapeutic application of drugs currently used to treat cardiovascular diseases to the treatment of patients with periodontal disease.

## Supporting Information

S1 Checklist ARRIVE Guidelines Checklist Animal Research: Reporting In Vivo Experiments. (DOC)

S1 Fig Blood pressure (mmHg) tracings from rats pretreated with drugs to test the effectiveness of drug doses. A relatively low (0.1 ng) or high dose (1.0 ng) of either angiotensin I (Ang I) or angiotensin II (Ang II) was injected into the femoral vein of rats given either water, enalapril, losartan, aliskiren via oral gavage for 14 days—time point of intravenous injection is indicated by black arrows. **A**) Rat pretreated with water. **B**) Rat pretreated with enalapril (10 mg/kg). **C**) Rat pretreated with losartan (50 mg/kg). **D**) Rat pretreated with aliskiren (30 mg/kg).
(TIF)

S2 Fig Inflammatory Cell Scoring of Rat Tissue Samples. One blinded researcher (COR) scored the inflammation of rats with 14 days of EP. Scores ranged from one through four and were based on inflammatory infiltrate, junctional epithelium, cementum and alveolar bone crest analysis. More specifically, tissue with a score of one had no inflammatory cellular infiltrate, preserved junctional epithelium, contained a few osteoclasts, and preserved alveolar processes and cementum. Tissue with a score of two had mild inflammatory cellular infiltrate, preserved junctional epithelium, contained some osteoclasts, minor alveolar process resorption and partial cementum destruction. A score of three indicated tissue with moderate inflammatory cellular infiltrate, apical migration of junctional epithelium, contained a large number of osteoclasts, moderate degradation of the alveolar processes, and partial cementum destruction. Tissue with a score of four had accentuated inflammatory cellular infiltrate, apical migration of junctional epithelium, contained a large number of osteoclasts and had severe resorption of alveolar processes and cementum.
(TIF)

S3 Fig qPCR of Renin-Angiotensin System Components in the Gingiva Tissue of Rats with Experimentally Induced Periodontitis. Results of qPCR analysis for mRNA of various RAS components extracted from the rat gingiva of either sham surgery (sham) or experimentally induced periodontitis (EP) for 14 days; tested RAS components included the following: angiotensinogen (AGT), angiotensin converting enzyme (ACE), angiotensin converting enzyme 2 (ACE-2), angiotensin II receptor type 1A (AT_1A_R), angiotensin II receptor type 1B (AT_1B_R) and angiotensin II receptor type 2 (AT_2_R). Each graph displays expression levels of the target mRNA relative to β-actin mRNA from 5 groups of 5 rats in duplicate given daily doses of water or drugs via gavage. Groups include the following: 14 d of water with sham surgery (sham), 14 d of water with experimentally induced periodontitis (water), 14 d of aliskiren (30 mg/kg) with experimentally induced periodontitis (aliskiren), 14 d of losartan (50 mg/kg) with experimentally induced periodontitis (losartan) or 14 d of enalapril (10 mg/kg) with experimentally induced periodontitis (enalapril). Bars represent the means with one SD. A one-way ANOVA and Tukey’s test with statistical significance set at *p*-value < 0.05 was used.
(TIF)
