# Supplementary material for: Functional Local Renin-Angiotensin System in Human and Rat Periodontal Tissue
Source: PLoS One. 2015 Aug 5;10(8):e0134601. doi: 10.1371/journal.pone.0134601 (PMC4526652; doi:10.1371/journal.pone.0134601)

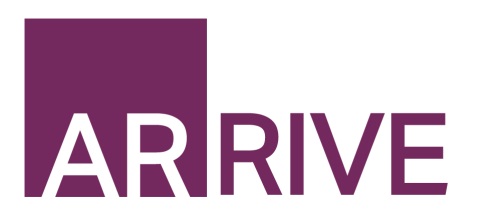


The ARRIVE Guidelines Checklist

Animal Research: Reporting In Vivo Experiments

Carol Kilkenny^1^, William J Browne^2^, Innes C Cuthill^3^, Michael Emerson^4^ and Douglas G Altman^5^

*^1^The National Centre for the Replacement, Refinement and Reduction of Animals in Research, London, UK, ^2^School of Veterinary Science, University of Bristol, Bristol, UK, ^3^School of Biological Sciences, University of Bristol, Bristol, UK, ^4^National Heart and Lung Institute, Imperial College London, UK, ^5^Centre for Statistics in Medicine, University of Oxford, Oxford, UK.*

|  | ITEM | RECOMMENDATION | Section/ Paragraph | |
| --- | --- | --- | --- | --- |
| 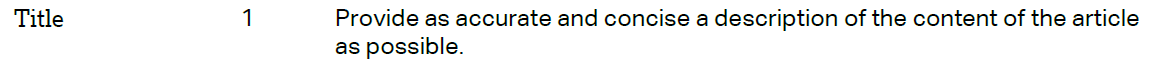 | | | Title | |
| 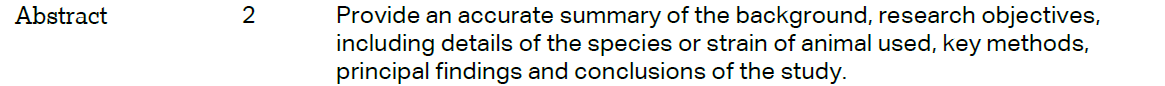 | | | Abstract | |
| INTRODUCTION | | |  | |
| 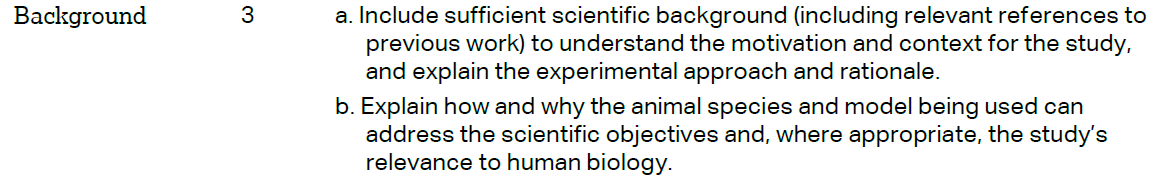 | | | a: Paragraphs 1-6  b: Paragraphs 1-6 | |
| 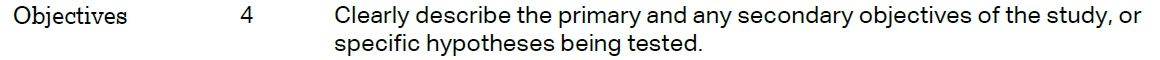 | | | Paragraph 6 | |
| METHODS | | |  | |
| 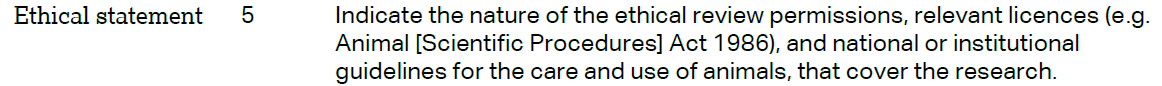 | | | Paragraph 1 | |
| 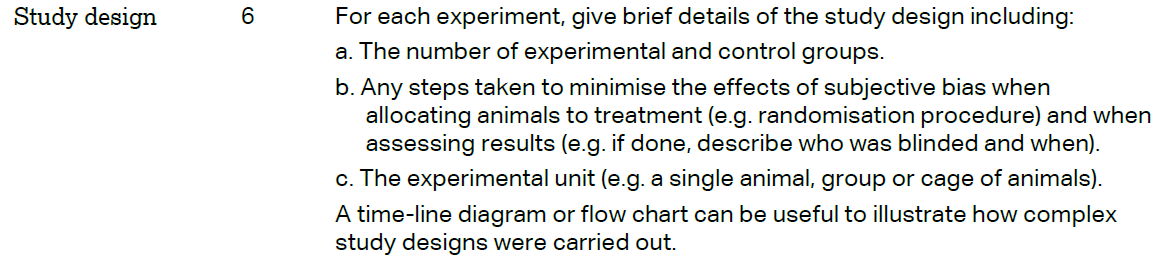 | | | a: Paragraphs 2-4,6-9,10,12,13, 15,16,19  b: Paragraphs 2-4,6,8,10, 12,14,15,17,18-20  c: Paragraphs 2-3 | |
| 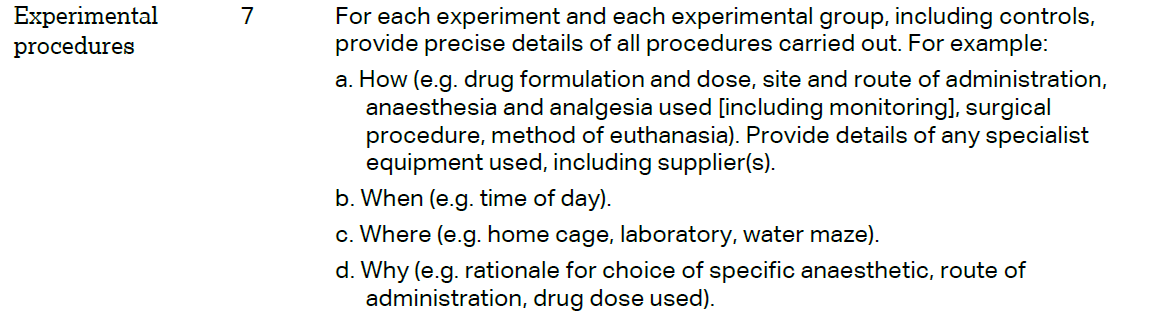 | | | a: Paragraphs 1,3,17  b: Paragraph 3  c: Paragraph 3  d: Paragraph 3 | |
| 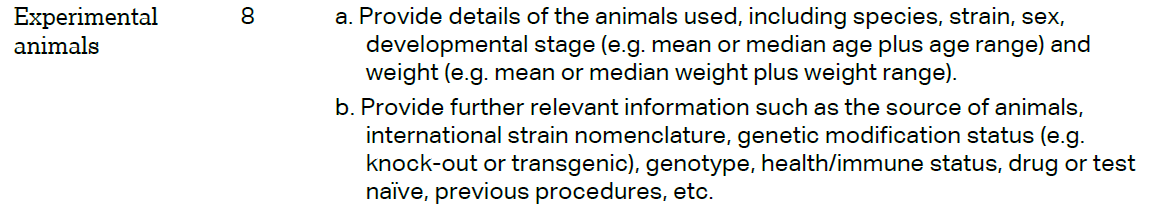 | | | a: Paragraph 3  b: Paragraph 3 | |
| 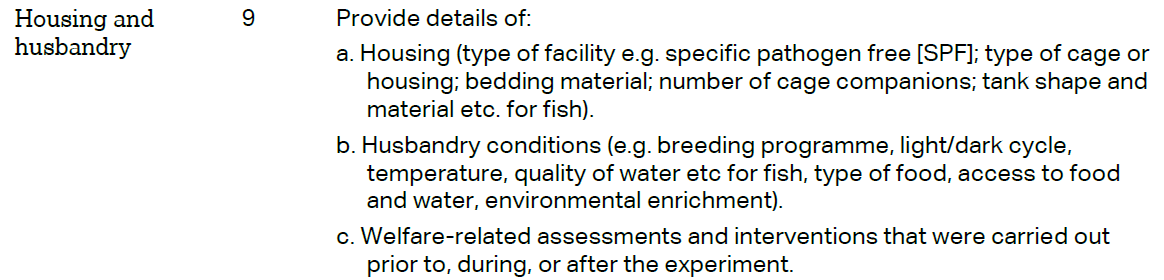 | | | a: Methods: Paragraph 3  b: N/A  c: N/A | |
| 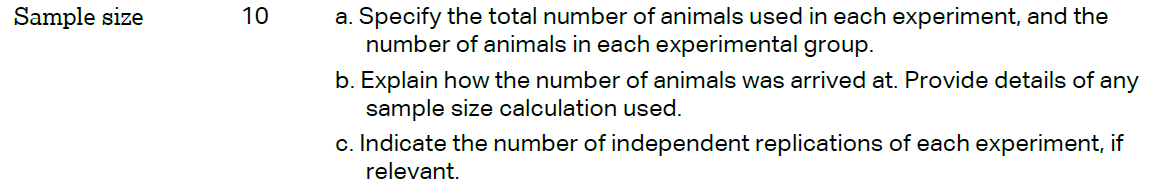 | | | a: Paragraphs 2-4,6,8,13, 14,19  b: Paragraph 3  c: Paragraphs 6,7,20 | |
| 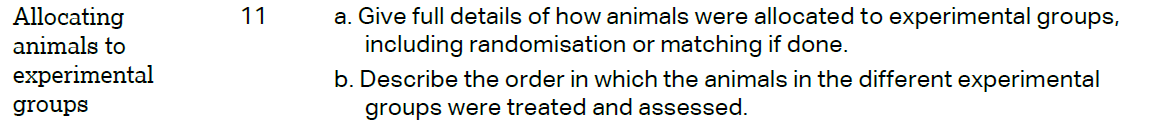 | | | a: Methods: Paragraph 3  b: Paragraph 3 | |
| 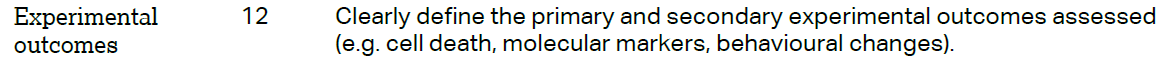 | | | N/A | |
| 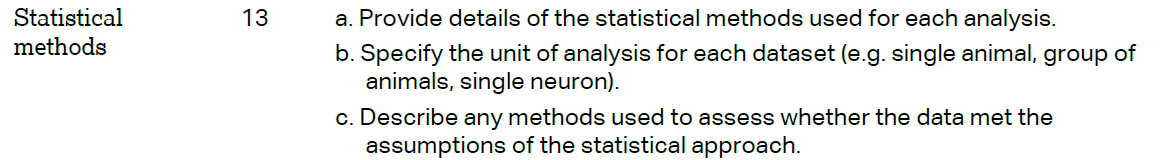 | | | a: Paragraph 22  b: Paragraph 37  c: Paragraphs 3, 22 | |
| RESULTS | | |  | |
| 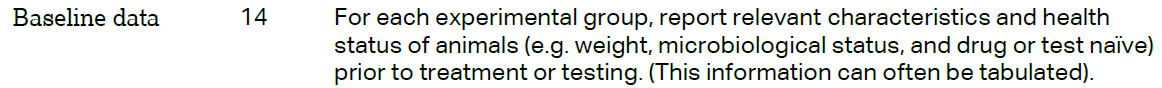 | | | Paragraph 1 | |
| 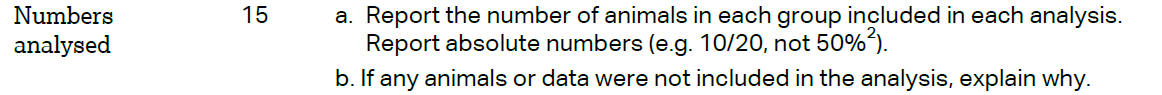 | | | a: Paragraph 8  b: N/A | |
| 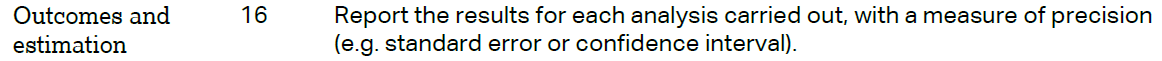 | | | Paragraph 2 | |
| 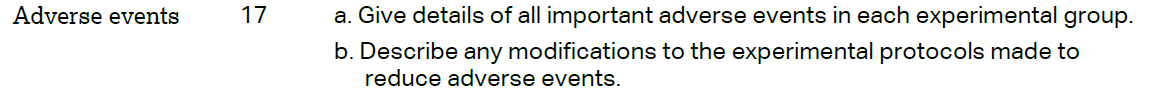 | | | a: N/A  b: N/A | |
| DISCUSSION | | |  | |
| 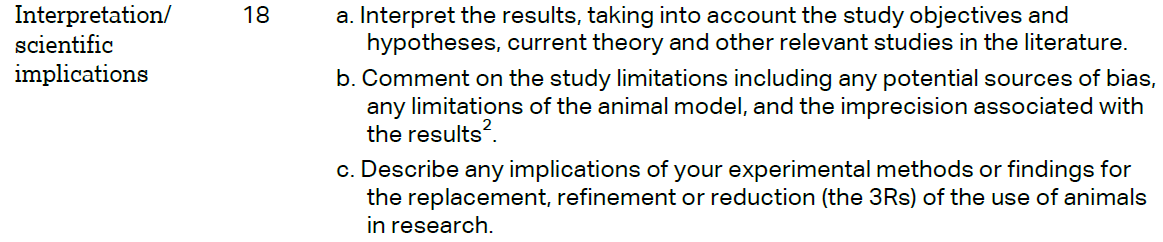 | | | a.  Paragraphs 1,3,5,7,8-15  b.  Paragraphs 2,4,6,9,10,  13 | |
| 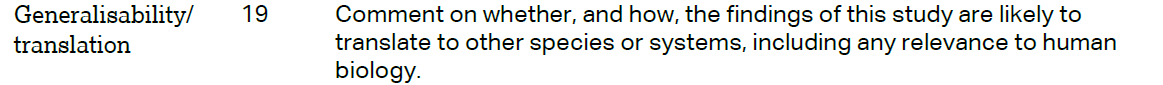 | | | Paragraphs 1-16 | |
| 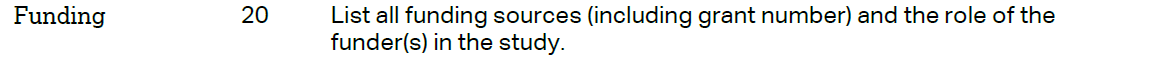 | | | Funding Sources |  |


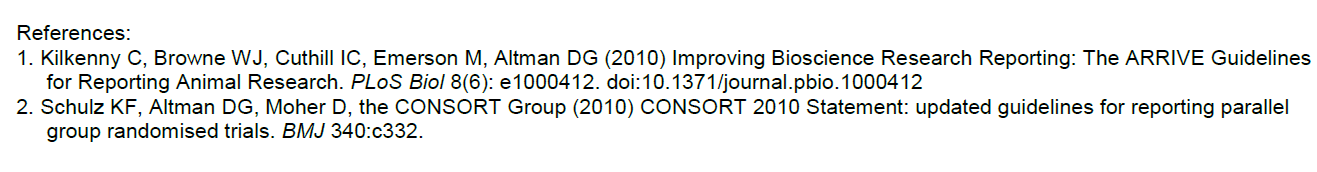

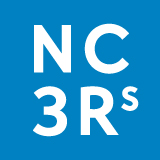

Supplement: S1 Checklist — (DOC) [file pone.0134601.s001.doc]
