# Supplementary figures and images for: Functional Local Renin-Angiotensin System in Human and Rat Periodontal Tissue
Source: PLoS One. 2015 Aug 5;10(8):e0134601. doi: 10.1371/journal.pone.0134601 (PMC4526652; doi:10.1371/journal.pone.0134601)

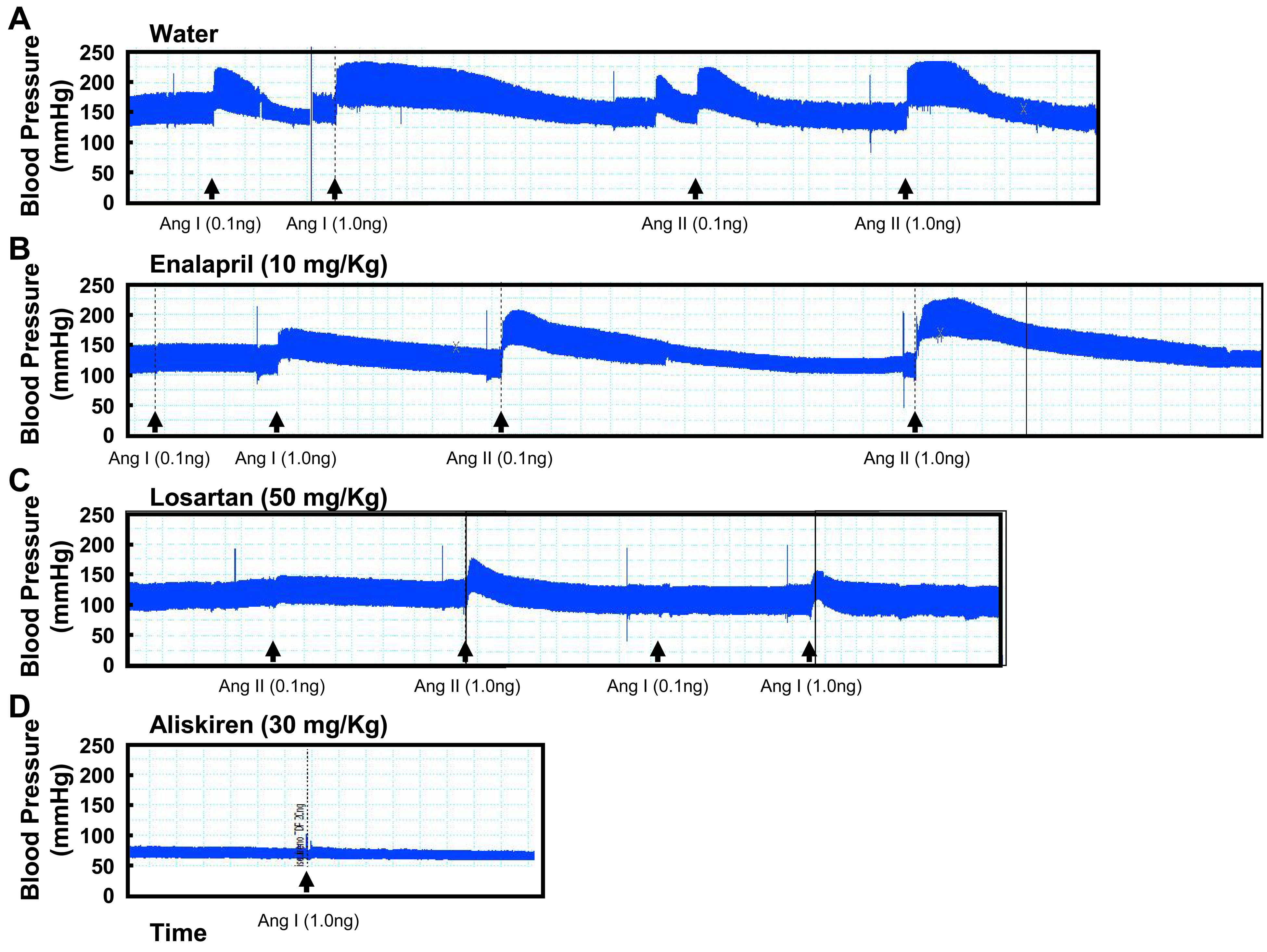

Supplement: S1 Fig — A relatively low (0.1 ng) or high dose (1.0 ng) of either angiotensin I (Ang I) or angiotensin II (Ang II) was injected into the femoral vein of rats given either water, enalapril, losartan, aliskiren via oral gavage for 14 days—time point of intravenous injection is indicated by black arrows. A) Rat pretreated with water. B) Rat pretreated with enalapril (10 mg/kg). C) Rat pretreated with losartan (50 mg/kg). D) Rat pretreated with aliskiren (30 mg/kg). (TIF) [file pone.0134601.s002.tif]

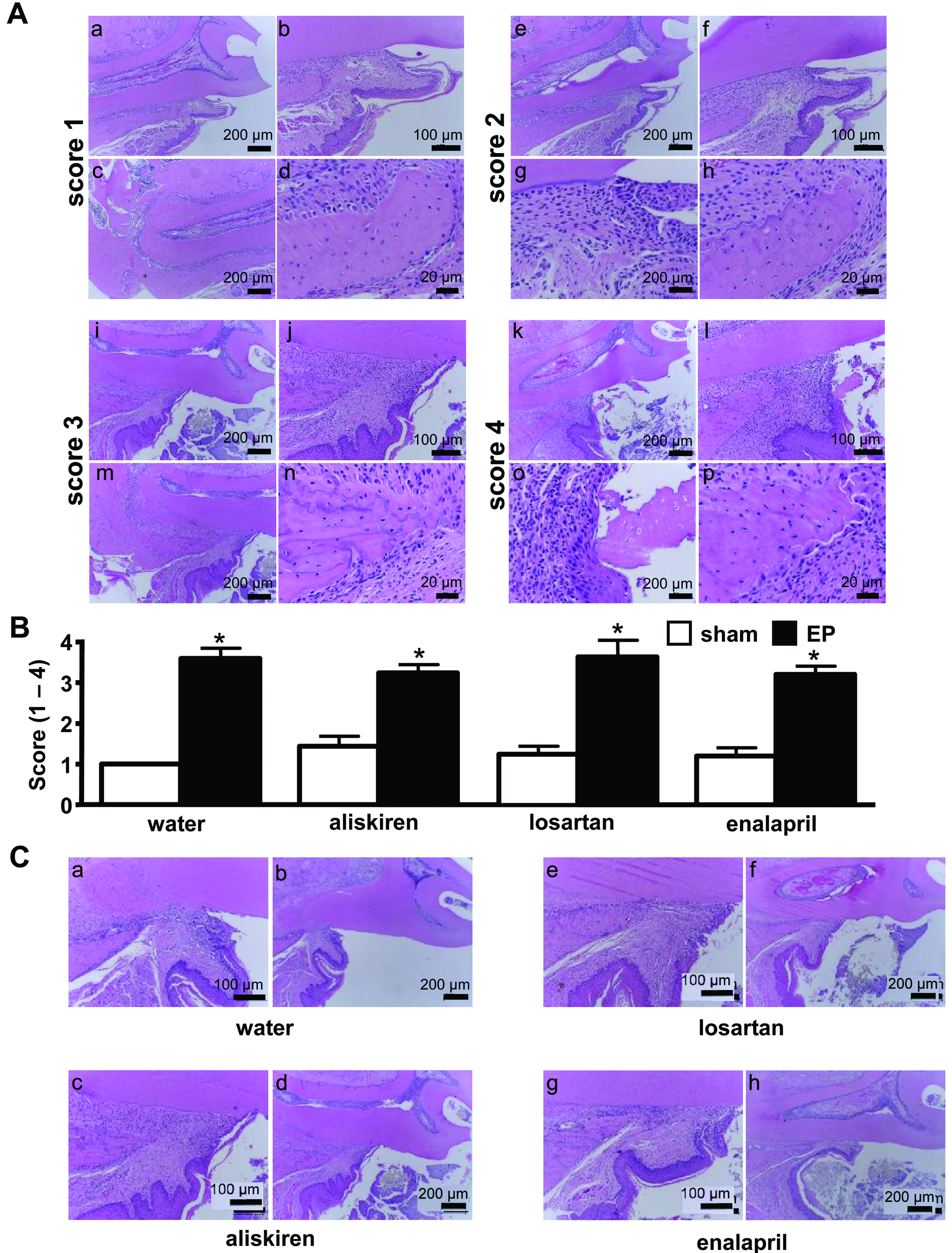

Supplement: S2 Fig — One blinded researcher (COR) scored the inflammation of rats with 14 days of EP. Scores ranged from one through four and were based on inflammatory infiltrate, junctional epithelium, cementum and alveolar bone crest analysis. More specifically, tissue with a score of one had no inflammatory cellular infiltrate, preserved junctional epithelium, contained a few osteoclasts, and preserved alveolar processes and cementum. Tissue with a score of two had mild inflammatory cellular infiltrate, preserved junctional epithelium, contained some osteoclasts, minor alveolar process resorption and partial cementum destruction. A score of three indicated tissue with moderate inflammatory cellular infiltrate, apical migration of junctional epithelium, contained a large number of osteoclasts, moderate degradation of the alveolar processes, and partial cementum destruction. Tissue with a score of four had accentuated inflammatory cellular infiltrate, apical migration of junctional epithelium, contained a large number of osteoclasts and had severe resorption of alveolar processes and cementum. (TIF) [file pone.0134601.s003.tif]

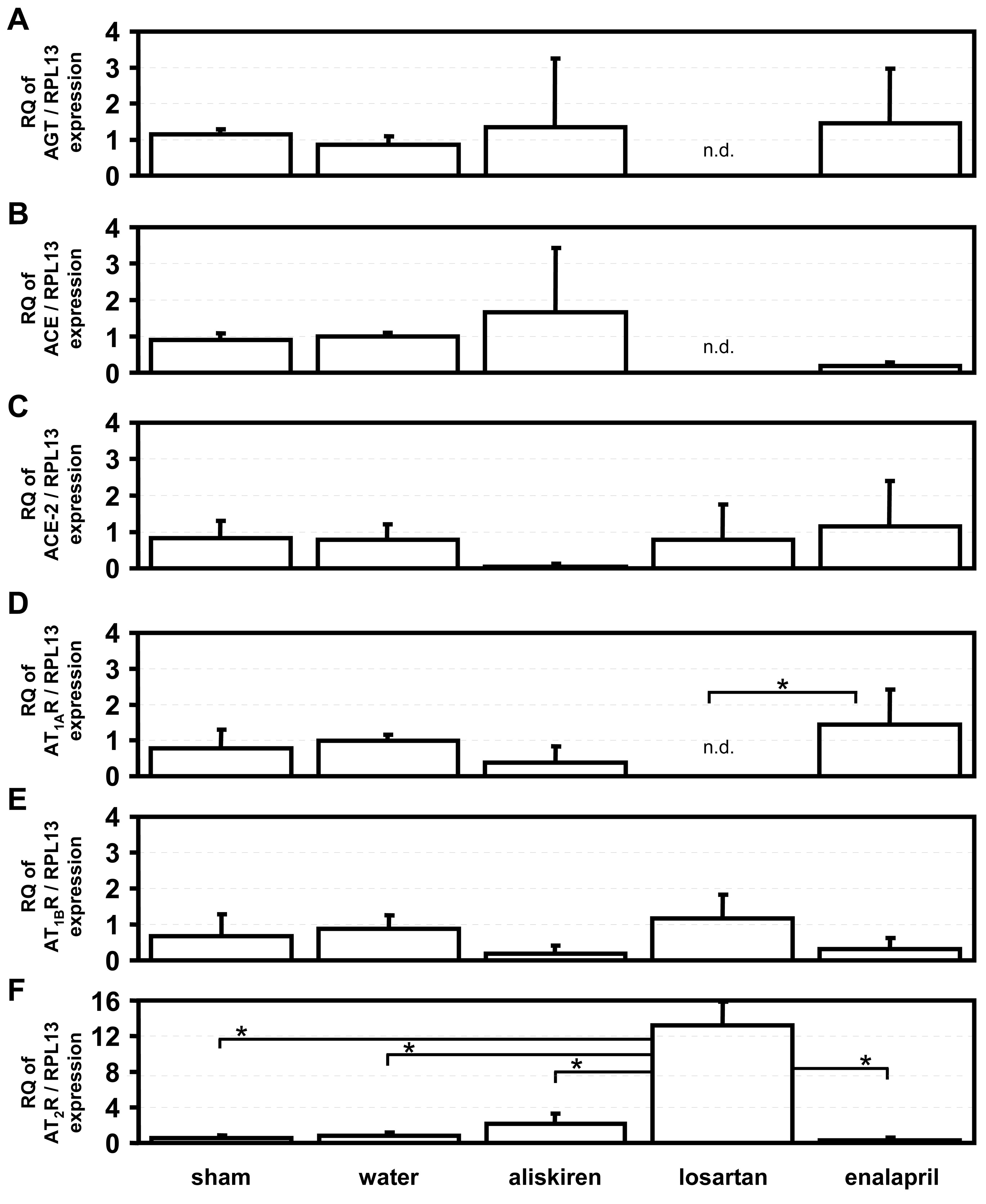

Supplement: S3 Fig — Results of qPCR analysis for mRNA of various RAS components extracted from the rat gingiva of either sham surgery (sham) or experimentally induced periodontitis (EP) for 14 days; tested RAS components included the following: angiotensinogen (AGT), angiotensin converting enzyme (ACE), angiotensin converting enzyme 2 (ACE-2), angiotensin II receptor type 1A (AT1AR), angiotensin II receptor type 1B (AT1BR) and angiotensin II receptor type 2 (AT2R). Each graph displays expression levels of the target mRNA relative to β-actin mRNA from 5 groups of 5 rats in duplicate given daily doses of water or drugs via gavage. Groups include the following: 14 d of water with sham surgery (sham), 14 d of water with experimentally induced periodontitis (water), 14 d of aliskiren (30 mg/kg) with experimentally induced periodontitis (aliskiren), 14 d of losartan (50 mg/kg) with experimentally induced periodontitis (losartan) or 14 d of enalapril (10 mg/kg) with experimentally induced periodontitis (enalapril). Bars represent the means with one SD. A one-way ANOVA and Tukey’s test with statistical significance set at p-value < 0.05 was used. (TIF) [file pone.0134601.s004.tif]
